# Gut Luminal Exosomes in Young and Old Mice: Multi‐Omic Characteristics and Regulation of Gut Permeability

**DOI:** 10.1111/acel.70455

**Published:** 2026-03-26

**Authors:** Abdelnaby Khalyfa, Lyu Zhen, Trupti Joshi, David Gozal

**Affiliations:** ^1^ Department of Biomedical Sciences, Joan C. Edwards School of Medicine Marshall University Huntington West Virginia USA; ^2^ Department of Electrical Engineering and Computer Science University of Missouri Columbia Missouri USA; ^3^ Department of Biomedical Informatics, Biostatistics and Medical Epidemiology University of Missouri Columbia Missouri USA; ^4^ MU Institute for Data Science and Informatics University of Missouri Columbia Missouri USA; ^5^ Christophers S. Bond Life Sciences Center University of Missouri Columbia Missouri USA; ^6^ Department of Pediatrics, Joan C. Edwards School of Medicine Marshall University Huntington West Virginia USA

**Keywords:** aging, extracellular vesicles (EVs), gut microbiome, gut permeability, insulin resistance, luminal fecal exosomes (LFEs), miRNAs, multi‐omics, proteomics

## Abstract

Aging is a multifaceted process impacting physiological, genomic, metabolic, and immune functions. This study investigates the role of luminal fecal exosomes (LFEs) in age‐associated metabolic dysfunction. We analyzed LFEs from young (3‐month) and old (24‐month) male and female C57BL/6 mice to characterize age‐related differences in exosomal proteomic and miRNA cargos. To explore interactions between LFEs and the gut microbiome, naïve young mice were gavage fed with LFEs from old donors, followed by 16S rRNA sequencing. Gut permeability in vitro and in vivo and systemic metabolic effects were assessed using ECIS, 3D microfluidic models, and insulin sensitivity assays. Bioinformatic analyses identified specific proteins and miRNAs linked to insulin resistance and barrier dysfunction. Heatmaps and principal component analysis revealed distinct differences in LFE profiles between young and old mice. Notably, LFEs from old mice impaired gut barrier integrity and metabolic function in young recipients, with reciprocal effects noted in older mice when receiving LFEs from young mice. Multi‐omics profiling, including proteomics and miRNA sequencing, identified age‐dependent and gender‐related changes in LFE cargo, encompassing host‐ and GM‐derived proteins and miRNAs. These age‐specific profiles were associated with pathways implicated in cancer, neurobehavioral changes, and metabolic dysfunction. Our findings highlight that LFEs from old mice are enriched with proteins and miRNAs involved in insulin resistance and gut barrier disruption. Together, these findings identify gut luminal exosomes as age‐dependent mediators of microbiome–host communication that contribute to intestinal barrier dysfunction and metabolic decline.

## Graphical abstract

This graphical abstract shows that gut luminal exosomes (LFEs) change with aging, with old mice producing LFEs that disrupt gut barrier integrity, alter microbiome composition, and promote insulin resistance. In contrast, LFEs from young mice improve gut barrier function and metabolic health, and transferring these LFEs can partially reverse age‐related gut and metabolic dysfunction.

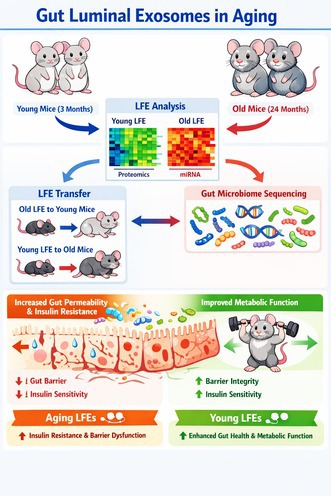

## 1 Introduction

Aging is a physiological process driven by biological and genetic pathways that regulate lifespan and disease susceptibility and is associated with progressive declines in immune function, as demonstrated in both murine and human studies (Hou et al. 2022; Li et al. 2021; Sun et al. 2023; Ukraintseva et al. 2021). Aging is a major risk factor for numerous chronic conditions, including Alzheimer's disease, cancer, cardiovascular disorders, and type 2 diabetes mellitus (T2DM), collectively referred to as age‐related diseases (Franceschi et al. 2018; Goldney et al. 2024; Guo et al. 2022; Hou et al. 2022; Niccoli and Partridge 2012). The global population is rapidly aging, and this clear epidemiological evidence is evoking substantial public health concerns. Indeed, by 2050, the global population aged ≥ 65 years is expected to more than double to 1.5 billion (Ghosh et al. 2022). The number of people over the age of 60 is expected to increase from 11% to about 22% by 2050 (Beard et al. 2016; Newgard and Sharpless 2013). Sex‐specific differences in lifespan and aging are widespread, with women generally living longer than men but experiencing greater frailty and different age‐related diseases (Fritz Garcia et al. 2024). Aging is also associated with widespread physiological changes, including dysbiosis of the gut microbiome (Lopez‐Otin et al. 2023). Age‐related alterations in gut microbial composition have been implicated in the development of multiple chronic diseases and contribute to disrupted host homeostasis, immune dysregulation, and chronic inflammation (Hou et al. 2022; Mitrea et al. 2022; Ragonnaud and Biragyn 2021; Xiao et al. 2025). Emerging evidence shows that alterations in gut microbial composition are associated with the pathogenesis of aging‐related human diseases (Dong and Mayer 2024; Fan and Pedersen 2021; Hall et al. 2017; Hou et al. 2022).

Accumulating evidence indicates that gut microbiome composition changes with age, including shifts in bacterial abundance and diversity (Ghosh et al. 2022; Haran and McCormick 2021; Hou et al. 2022; Odamaki et al. 2016). Although comprehensive characterization of the trillions of microorganisms and millions of genes that comprise the gut microbiome remains challenging, its composition is continuously shaped by host factors such as the intestinal environment, diet, physical activity, circadian rhythms, and age (Hou et al. 2022; Li et al. 2014; Qin et al. 2010; Rajilic‐Stojanovic and de Vos 2014). The intestinal microenvironment plays essential roles in nutrient metabolism, organ development, and immune regulation, and disruptions in microbial balance can impair host metabolic and immune functions, ultimately contributing to disease (Clemente et al. 2012; Routy et al. 2018; Zeng et al. 2017). The gut microbiome changes with age, shifting in both microorganisms and function, and also differs by sex, with adult patterns shaped in part by sex hormones that microbes help metabolize (Kim et al. 2020; Santos‐Marcos et al. 2019; Yoon and Kim 2021).

Extracellular vesicles (EVs) are nano‐sized particles released by most cell types and play a critical role in intercellular communication and diverse biological processes (Couch et al. 2021). EVs carry a complex cargo of lipids, nucleic acids, and proteins, including components associated with the plasma membrane, cytosol, and lipid metabolism (Yáñez‐Mó et al. 2015). Through the transfer of this cargo, EVs influences cellular signaling and physiological homeostasis, making EVs promising candidates for therapeutic applications, drug delivery systems, and disease biomarkers (Cuesta et al. 2021). Recent studies have demonstrated that microbial EVs modulate the intestinal microenvironment and host health (Diaz‐Garrido et al. 2021; Nishiyama et al. 2020). Among these, bacterial extracellular vesicles (BEVs) represent an important mechanism of communication, as they can interact with other bacterial cells as well as host cells (Peregrino et al. 2024). BEVs secreted by gut commensal, probiotic, and pathogenic bacteria have been shown to exert regulatory effects and may cross biological barriers, including entry into the bloodstream or central nervous system (CNS) (Liang et al. 2022; Turunen et al. 2023). Several subtypes of BEVs, commonly referred to as membrane vesicles (MVs), have been identified and are distinguished by their membrane origin and mode of release. These vesicles have been described in both Gram‐positive and Gram‐negative bacteria (Jahromi and Fuhrmann 2021; Nagakubo et al. 2019). BEVs, which are similar in size to mammalian EVs, have been isolated from a range of in vitro and in vivo sources and are thought to mediate host–bacteria communication by carrying proteins, nucleic acids, lipids, and metabolites (Liu et al. 2018; Nahui Palomino et al. 2021). BEVs mediate communication between bacteria and their hosts through interactions with host receptors, delivery of EV cargo, and full incorporation into the host cell cytoplasm (Kawagishi et al. 2025; Liu et al. 2018; O'Donoghue and Krachler 2016; Qian et al. 2025).

Transferring fecal microbiota transplantation (FMT) from healthy human hosts to individuals with metabolic syndrome increased GM diversity and improved insulin sensitivity (Koren et al. 2016), and FMT from lean healthy individuals into obese recipients improved insulin sensitivity and blood pressure (BP) (Del Chierico et al. 2021; Vrieze et al. 2012). We and others have found that chronic IH mimicking sleep apnea induces pronounced changes in GM (Khalyfa et al. 2021; Lucking et al. 2018; Moreno‐Indias et al. 2016; Tripathi et al. 2019), and such changes lead to systemic inflammation and metabolic dysfunction (Barcelo et al. 2016; Gileles‐Hillel, Almendros, et al. 2014; Gileles‐Hillel, Alonso‐Alvarez, et al. 2014). Previously, we showed that FMT from mice exposed to a model of sleep apnea to naïve mice induced sleep disturbances, metabolic dysfunction, and white adipose tissue inflammation (Badran et al. 2020; Khalyfa et al. 2021; Poroyko et al. 2016). It is likely that changes in the gut barrier constitute the first portal of entry allowing for bacteria to enter systemically, causing inflammation and metabolic disorders (Paone and Cani 2020; Stange and Schroeder 2019). FMT from healthy young donors restored gut permeability and several other functions in aged mice, including animals suffering from aging‐related disorders (Novelle et al. 2025; Zheng et al. 2022). For example, FMT from young healthy female mice to middle‐aged mice exposed to alcohol significantly reduced liver injury and inflammation as well as reduced intestinal permeability (Lamas‐Paz et al. 2024).

We hypothesized that aging alters the molecular cargo of gut luminal exosomes, thereby impairing intestinal barrier integrity and systemic metabolic regulation. To test this hypothesis, we aimed to examine the effects of age‐associated microbiota composition on extracellular vesicles cargos and the effects of such EVs on gut permeability using multi‐modality approaches (Dayan et al. 2017; Kiseleva et al. 2024). Therefore, we conducted young‐ and age mice male and female (i) to characterize LFEs and their effects on gut barrier integrity; (ii) to evaluate the impact of LFEs transplantation from young and old mice on gut microbiota diversity and composition; (iii) to characterize LFEs cargos including proteomics for both host and bacteria; as well as LFEs miRNAs to provide rapid mechanistic insights into how LFEs from young mice may benefit the intestinal inflammation of aging.

## 2 Materials and Methods

### 2.1 Animals

Male and female C57BL/6J mice used in these experiments included young (3 month‐old, *n* = 12), and old animals (24 month‐old, *n* = 12) which were purchased from Jackson Laboratory (Bar Harbor, ME, USA). The mice were kept continuously under controlled conditions of lighting (12‐h light/dark cycle, lights on at 6:00 am), ambient temperature (23°C–24°C ± 1.0°C), and humidity (40%–70%) in the vivarium facility with free unrestricted access to food and water. All procedures performed were approved by University of Missouri Health Institutional Animal Care and Use Committee (IACUC). At the end of each experiment, mice were euthanized using CO_2_ exposures followed by cervical dislocation. Organs were harvested, and blood was collected in EDTA‐containing tubes and immediately centrifuged with plasma samples being stored at −80°C until assay.

### 2.2 Luminal Fecal Exosomes (LFEs) Isolation

Fecal samples (250 mg) were collected at the same time of the day at 1 pm and immediately stored at −80°C. Fecal samples from each animal were mixed and suspended in 6.5 mL PBS by extensive vortex. Disrupted pellets were centrifuged at 500×*g* for 10 min to precipitate insoluble materials, to which 6.5 mL PBS were added to re‐suspend the pellets. This step was repeated twice. The suspension was then combined and centrifuged at 2000*g* for 10 min to pellet bacteria. This nominally bacteria‐free supernatant was further filtered through 40 μm and further filtered through 0.22 μm filters to further remove any bacteria. The supernatants were concentrated using Amicon Ultra Centrifugal Filter, 30 kDa MWCO (Sigma‐Aldrich Inc., St. Louis, MO). The feces supernatants were centrifuged at 150,000×*g* for 2 h using an ultracentrifuge (Beckman Coulter, OptimaTMMAX‐XP, USA) with a TYPE 45 Ti swing rotor. The pellet was collected by pipetting the supernatant, resuspended in PBS, and then centrifuged at 150,000 × g for another 2 h. The pellets of LEVs were resuspended in PBS for analysis. All centrifugations were performed at 4°C. The pellets were solubilized in filtered 1xPBS. The samples were stored at −20°C for 3–4 weeks and at −80°C for long‐term storage. The isolated LFEs were subsequently quantified and characterized following MISEV2018 guidelines (Thery et al. 2018).

### 2.3 LFEs Characterization

#### 2.3.1 Transmission Electron Microscopy

Isolated LFEs solutions were diluted 1:1000 in Dulbecco's Phosphate Buffered Saline, DPBS (Life Technologies, Grand Island, NY, USA), and 7 μL of diluted exosomes were placed on parafilm before the Formvar/Carbon‐coated grid was placed on top of exosome drops and allowed to stand for 2 min. Grids with adherent exosomes were washed three times with 25 μL DPBS drops and fixed with 2% paraformaldehyde in DPBS for 7 min. Finally, grids were incubated with 25 μL drops of 2% uranyl acetate and examined by electron microscopy. The samples were washed with distilled water seven times (2 min each), and then they were viewed under a FEI Tecnai F30 microscope.

#### 2.3.2 Flow Cytometry

Purified LFEs were labeled by incubation with Exo‐Flow kits (System Biosciences, Mountain View, CA, USA) and then subjected to FACS analysis (FACSCalibur, BD Biosciences, San Jose, CA, USA). Briefly, LFEs were incubated with commercially available magnetic beads of 9.1 nm diameter that incorporated different EV markers including tetraspanins (CD63). LFEs and the magnetic beads were incubated for 12 h at 4°C according to the manufacturer's manual. Two negative controls were also included, with negative #1 (all reagents without antibodies and no EVs) and negative #2 (all the reagents and beads but without EVs). In the FACS, 40,000 events were acquired and then analyzed using FlowJo Software (Tree Star Inc., Ashland, OR, USA). The average MFI for negative #1 was used to normalize the samples with and without EVs.

### 2.4 LFEs Uptake

LFEs were labeled with PKH26 (Sigma‐Aldrich) at 37°C for 10 min, followed by precipitation using ExoQuick‐TC reagents (System Biosciences). The mixture was then incubated on ice for 30 min, centrifuged at 4°C at 13,000 rpm, and filtered to remove any unbound dyes. The resulting pellets were resuspended in 1× PBS buffer. Labeled LFEs were then added to confluent coverslips of murine primary small intestinal epithelial cells (Cell Biologics, Chicago, IL) and incubated for 24 h in a cell culture incubator at 37°C. Imaging of the PKH26‐Red label was conducted using a confocal microscope (Leica Microsystems Inc., Buffalo Grove, IL, USA) equipped with a 63× oil‐immersion lens. A negative control was prepared by labeling PKH26‐Red with all reagents without LFEs to check for any unincorporated dyes that may have carried over during centrifugation. Nuclei were visualized using Hoechst 33,342 (Sigma‐Aldrich) at a concentration of 1 μg/mL in PBS for 5 min. The mean PKH26 intensity per cell was determined using ImageJ/Fiji: draw cell ROI.

### 2.5 Permeability and Barrier Integrity Measurements In Vitro

#### 2.5.1 Electric Cell–Substrate Impedance Sensing (ECIS)

Murine primary small intestinal epithelial cell monolayer integrity was assessed using the Electric Cell–Substrate Impedance Sensing (ECIS) system. ECIS measures electrical impedance across 250‐μm gold electrodes that serve as substrates for cell growth. PC (8W10E) arrays were pretreated with 10 mM L‐cysteine. A total of 75,000 cells were plated per well and allowed to reach confluence over 24 h, forming a single monolayer. LFEs (5 μg protein) were then added in epithelial cell medium supplemented with 10% depleted fetal bovine serum, and impedance was monitored continuously for an additional 24 h. Capacitance was measured at 40 kHz and resistance at 400 Hz to assess changes in barrier integrity associated with cell adhesion and junctional disruption. Control values were obtained from medium alone and compared with measurements from cell‐covered electrodes (Khalyfa et al. 2021).

#### 2.5.2 Gut Permeability In Vitro

The OrganoPlate 3‐lane platform (4004‐400B; MIMETAS BV, Netherlands) contains a central gel channel flanked by two perfusion channels, separated by PhaseGuides that enable gel and cell patterning without artificial membranes. Each channel connects to inlet and outlet wells in the microtiter plate, allowing compartmentalized culture and gradient formation. An extracellular matrix was established by loading 2 μL of collagen I–based gel (4 mg/mL collagen I, 100 mM HEPES, and 3.7 mg/mL Na_2_HCO_3_) into the central channel and incubating for 30 min at 37°C. Murine small intestinal epithelial cells (2 μL) were seeded into one perfusion channel, and the plate was inverted and incubated for 1 h to allow cell attachment. Medium was then added, and bidirectional flow was applied using an OrganoFlow system (±7° every 8 min). Non‐adherent cells were flushed out, and cultures were maintained for 6 days with daily medium changes. On Day 6, luminal fecal exosomes (2 μg protein) were applied for 24 h. Barrier permeability was then assessed by adding 20 kDa FITC–dextran (0.1 mg/mL) to the luminal channel. After 10 min, dextran leakage into the gel channel was imaged at 4× magnification using a Nikon microscope. Permeability was quantified in Fiji (ImageJ) as the fluorescence ratio between apical and basal regions. Blank chips without cells served as controls.

### 2.6 Gut Permeability In Vivo

Young and old male and female mice were fasted for 4 h, and approximately 50 μL of blood was collected from the tail vein to establish baseline (time zero) values. FITC‐dextran (4 kDa; Sigma‐Aldrich, St. Louis, MO) was prepared at 80 mg/mL in phosphate‐buffered saline (PBS), and 150 μL was administered to each mouse by oral gavage (*n* = 6–8). Blood samples were collected for up to 4 h following gavage. Plasma was isolated by centrifugation at 2000×*g* for 5 min and diluted 1:10 (v/v) in PBS for analysis. Fluorescence was measured in duplicate using a photomultiplier tube–based detection system (GloMax‐Multi Detection System; Promega, Madison, WI) in 96‐well plates (excitation: 485 nm; emission: 528 nm). Values were averaged, and intestinal permeability was expressed as relative fluorescence units for the groups compared. This method is a noninvasive technique used to quantify and monitor intestinal permeability in mice in real time using fluorescein isothiocyanate–labeled dextran (FITC‐dextran).

### 2.7 Gavage Feeding of LFEs From Old Mice Into Young Naïve Mice

The LFEs from old mice were prepared as described above, and 30 ug of protein (100 μL) per mouse was gavage fed three times a week for 8 weeks into a naïve C57BL/6 mice 8‐weeks old. At the end of this period, feces, plasma, and organs were collected for further analysis.

### 2.8 Glucose and Insulin Tolerance Tests (GTTand ITT)

Mice treated with LFEs for 8 weeks were subjected to a glucose tolerance test (GTT) after 3 h of fasting, during which water was freely available. Following the fasting period, glucose (2 mg/g body weight) was administered intraperitoneally. Blood samples for glucose determination were collected from the tail vein using heparin‐coated capillary tubes at 0, 4,15,30,60, and 120 min post‐injection. The glycemic response during the GTT was evaluated by calculating the total area under the glycemic curve as a function of time as previously reported (Gozal et al. 2016).

ITT was performed in a separate group of mice treated with LFEs. Mice were injected intraperitoneally with Humulin (0.25 U/kg body weight) after 3 h of fasting at the conclusion of the 8‐week LFEs gavage treatment. Blood glucose levels were measured at 0, 15, 30, 60, 90, and 120 min post‐injection using a OneTouch Ultra2 glucometer (Life Scan Inc., Milpitas, CA, USA). Additional blood samples for insulin measurement were collected from the cut tip of the tail. Glycemic trajectories from the ITT were analyzed to assess insulin sensitivity, as previously described (Carreras et al. 2012; Gozal et al. 2016). The area under the curve (AUC) was calculated as previously described (Badran et al. 2024; Sakaguchi et al. 2016).

### 2.9 Small Intestine Cell Culture

Murine primary small intestinal epithelial cells were grown as described above. Cells were further grown in 12‐well plates until they reached confluency and then cell media was replaced with depleted FBS and treated with LFEs for 24 h. in a humidified atmosphere of 5% CO_2_ at 37°C.

### 2.10 Gut Microbiome Profiling by 16S rRNA Sequencing

Feces from mice gavage‐fed with LFEs for 8 weeks were processed using PowerFecal kits (Qiagen) according to the manufacturer's instructions (Johnson et al. 2018; Khalyfa et al. 2017; Poroyko et al. 2016). Fresh stool samples were collected for each individual mouse and immediately snap‐frozen in liquid nitrogen before storage at −80°C until DNA extraction. Briefly, bacterial 16S rRNA amplicons were constructed via amplification of the V4 region of the 16S rRNA gene with universal primers (U515F/806R) as previously developed against the V4 region, flanked by Illumina standard adapter sequences (Johnson et al. 2018). The final amplicon pool was evaluated using the Advanced Analytical Fragment Analyzer automated electrophoresis system, quantified using quant‐iT HS dsDNA reagent kits, and further diluted according to Illumina's standard protocol for sequencing on the MiSeq instrument. The Illumina sequencing adapters and dual‐index barcodes are added to the amplicon target using the Nextera XT Index Kit (Illumina), according to the manufacturer's instructions. The amplicons were sequenced on a MiSeq (Illumina) according to the manufacturer's recommendations. Multivariate statistical analyses such as ANOVA and volcano plots were performed with the MetaboAnalyst 6.0 program after data pre‐treatments, that is, normalization to the sum, log transformation and Pareto scaling (Pang et al. 2024).

### 2.11 Exosome Proteomics

#### 2.11.1 In‐Solution Digestion

Proteins were extracted from young and old (male and female) LFE samples using RIPA buffer containing protease inhibitors. Total protein was quantified by BCA assay, while OSA and OSAT protein concentrations were measured by Bradford assay. For proteomic analysis, 75 μg of exosome protein per sample was precipitated with cold acetone overnight at −20°C, washed with 80% acetone, and resuspended in urea buffer. Proteins were digested with LysC followed by overnight trypsin digestion. Peptides were purified using C18 tips, lyophilized, resuspended in 5% acetonitrile/0.1% formic acid, and stored at 7°C. Peptides were analyzed by nanoLC–MS/MS using a Bruker nanoElute system coupled to a timsTOF Pro mass spectrometer. Samples were trapped on a C8 column and separated on a C18 analytical column using an acetonitrile gradient at 300 nL/min. PASEF LC–MS data were acquired and analyzed with PEAKS X‐Pro against the UniProt Mouse database. Searches allowed two missed cleavages, with carbamidomethylation as a fixed modification and oxidation and deamidation as variable modifications. Data were filtered at < 0.1% peptide FDR, requiring at least one unique peptide per protein. Quantitation was based on precursor ion intensity with mass and retention time correction. Protein interaction predictions were performed using the STRING database (v11.0) (Szklarczyk et al. 2019).

### 2.12 LFEs miRNAs

Total RNAs, including miRNAs, were isolated from exosomes using the miRNeasy Plasma mini‐Kit column (Qiagen, Turnberry Lane, Valencia, CA) as described (Khalyfa et al. 2021, 2020, 2018). The RNA quality and integrity were determined using the Eukaryote Total RNA PicoChip 6000 LabChip assay (Agilent) on the Agilent 2100 Bioanalyzer. The quality of miRNAs was determined using the Agilent Small RNA Kit.

#### 2.12.1 Whole Genome miRNA Sequencing

We constructed a miRNA library with an NEBNext Multiplex Small RNA Library Prep Set for Illumina (NEB, Ipswich, USA) according to the manufacturer's instructions. Briefly, total RNAs, including miRNAs (100 ng), were utilized. 3′ and 5′ RNA adapters will be ligated to RNAs by T4 RNA ligase. cDNAs were constructed by reverse transcription from the adapter ligated RNAs and amplified by 13 cycles of PCR. For the size selection of amplified cDNA libraries, PCR products were run on a 6% TBE gel with a custom ladder. The small RNAs, 140–160 base pairs, were excised, incubated overnight, and eluted using a spin column. The resulting libraries were subjected to the Illumina NovaSeq sequencing platform with 50 nucleotides single reads of 20 million reads per sample. Sequence quality control (FASTQC) was followed by alignment to the mouse genome (grch38) and mapped by miRBase_v.21.0. Reads with a Phred score < 20 were trimmed using the SolexaQA++ v3.1.7.1 (Burrows‐Wheeler Aligner trimming algorithm). Reads with lengths shorter than 17 bases were discarded, and the remaining reads were aligned to the mouse genome (GRCm39) using miRDeep2 v0.0.8 (Bowtie algorithm). miRNAs were predicted using the default settings of miRDeep2 and known miRNAs (miRBase, release 22) as reference sequences. Small RNA annotation was performed using the Unitas pipeline to remove non‐miRNA sequences. mirPRo and miRDeep2 were used to quantify known miRNAs and predict putative miRNAs. Reads were collapsed into a set of unique reads and aligned to Ensembl mouse genome and miRNAs sequences (miRBase, release 22).

#### 2.12.2 Target Predictions and Functional Annotation

Gene targets for differentially expressed miRNAs were initially computationally predicted using established miRWalk target‐prediction software (Sticht et al. 2018). Provided gene targets were uploaded to the online gprofiler (Kolberg et al. 2023; Reimand et al. 2007) for functional annotation and clustering analysis. Genes based on their associated gene ontology annotations, and the related terms, were clustered into groups with enrichment scores calculated from their EASE Score and the modified Fisher exact *p* value (Dennis 2003). The web server hosts a continuously updated version of the Kyoto Encyclopedia of Genes and Genomes (KEGG) database release 94.0, which provided a relevant search module based on KEGG pathway descriptions.

### 2.13 Multi‐Omics Integrative Analysis

A comprehensive integrative analysis of multi‐omics data was conducted using the mixOmics R package (Rohart et al. 2017). The datasets included proteomic and miRNAs profiles collected from young and old mice for both male and female mice. Prior to analysis, each dataset was preprocessed to ensure quality and consistency. This included normalization, missing value imputation, and log‐transformation, as necessary. We employed the mixOmics framework to conduct statistical analyses to identify biomarkers across different omics layers. We applied multivariate statistical tests to assess the significance of the identified variables, with a threshold set at *p* < 0.05 for determining statistical significance. The integration process used the Data Integration Analysis for Biomarker Discovery (DIABLO) framework, implemented in the mixOmics package (v6.10.9) (Singh et al. 2019).

### 2.14 Statistical Analysis and Sample Size

Statistical analyses were performed using GraphPad Prism version 10.2.3 (GraphPad Software, La Jolla, CA, USA) and SAS version 9.4 (SAS Institute, Cary, NC). Data are presented as mean ± standard deviation (SD). Comparisons between two groups were conducted using unpaired *t*‐tests for normally distributed data or Mann–Whitney U tests for nonparametric data. Comparisons among more than two groups were analyzed using Two‐way ANOVA followed by Tukey's post hoc test, or Kruskal–Wallis tests for nonparametric data. Differential gene abundance was evaluated using two‐tailed Wilcoxon tests for median comparisons, with Benjamini–Hochberg false discovery rate correction applied to adjust q‐values for multiple testing. To handle missing data in the integrative analysis, we applied a two‐step approach within the mixOmics framework. First, features with more than 20% missing values across samples were filtered out to ensure data robustness. For the remaining missing entries, we employed the NIPALS (Non‐linear Iterative Partial Least Squares) algorithm—built into the mixOmics package—which allows for the estimation of missing values during the decomposition of the data matrices. Alternatively, for datasets with low‐frequency missingness, K‐Nearest Neighbors (KNN) imputation was applied to the normalized intensity/count matrices to create a complete data structure for multivariate analysis. All analyses were conducted in a randomized and blind manner. A *p*‐value of ≤ 0.05 was considered statistically significant. Based on our previous studies, a sample size of 6–8 per group was sufficient to detect statistically significant differences (*p* < 0.05).

## 3 Results

### 3.1 Luminal Fecal Extracellular Vesicles Characterization

**FIGURE 1:**
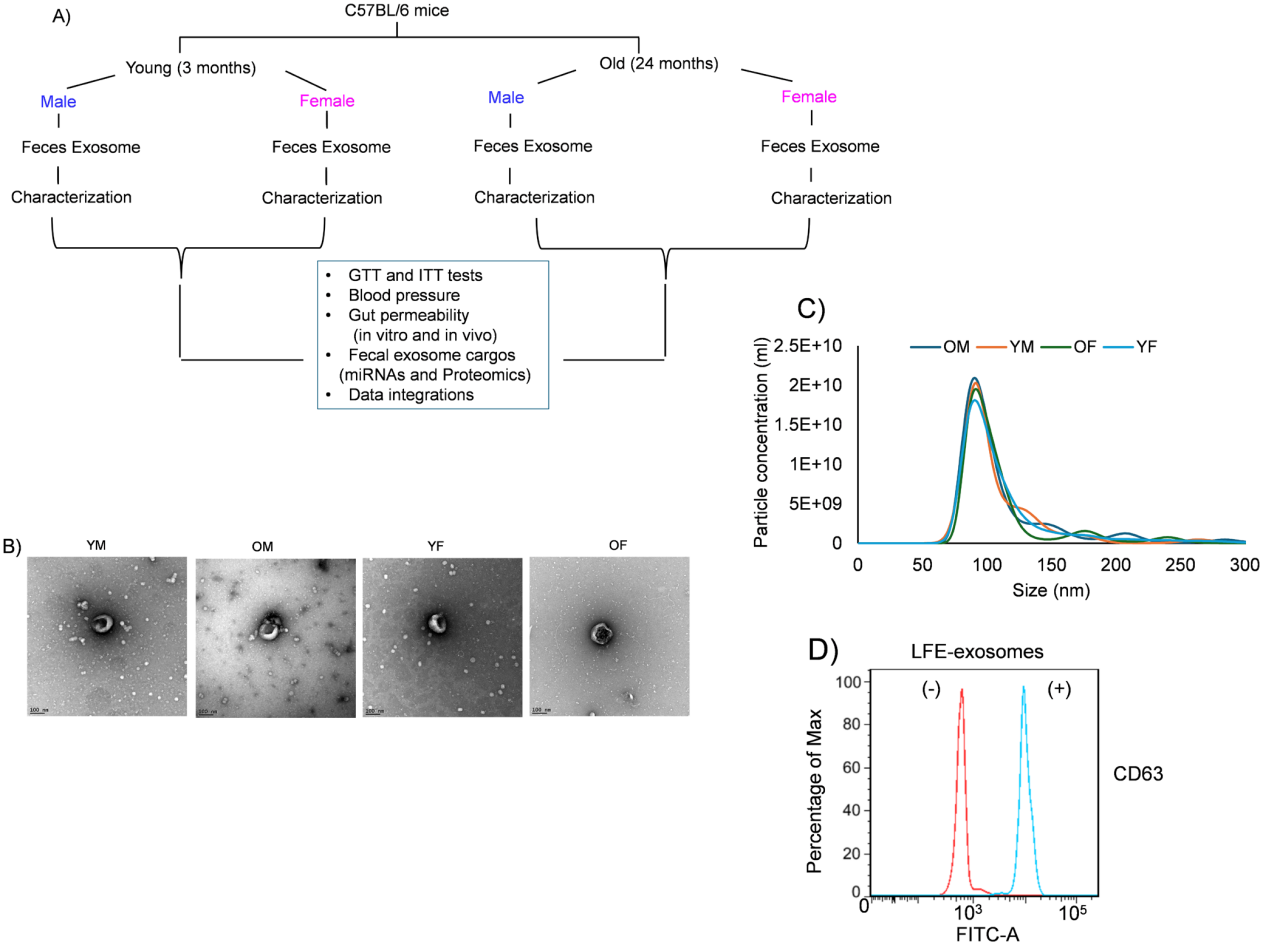
Experimental design and characterization of luminal fecal exosomes (LFEs). Fecal samples were collected from young (3‐month‐old) and old (24‐month‐old) male and female mice, followed by LFE isolation and quantification. (A) Schematic of the experimental design showing age‐ and sex‐based grouping and downstream multi‐omics analyses. Mice were assigned to young male (YM), old male (OM), young female (YF), and old female (OF) groups. (B) Representative transmission electron microscopy (TEM) images of LFEs. (C) Nanoparticle tracking analysis (NTA) for LFE size distribution and concentration. (D) Flow cytometry analysis of purified LFEs stained with anti‐CD63. Sample size was *n* = 6–8 mice per group.

To assess the impact of luminal fecal exosomes (LFEs) on young (3‐month‐old) and old (24‐month‐old) male and female mice, we designed an experimental framework to evaluate age‐ and sex‐dependent effects on host multi‐omics profiles (Figure 1A). This experimental design outlines the grouping of mice by age and sex and the subsequent integration of downstream multi‐omics analyses to characterize LFE‐associated molecular changes.

LFEs were characterized by using electron microscopy (EM) for size morphology, quantification, and protein markers encompassing tetraspanin proteins (Figure 1B–1D). The LFEs' shape and expression of the exosomal marker were confirmed by negative staining of EM (Figure 1B), and the correct size range (30–150 nm) was assessed by NTA (Figure 1C). The average size of LFEs in YM was (2.01 × 10^10^), OM (2.06 × 10^10^), YF (1.81 × 10^10^), and OF (1.95 × 10^10^), respectively (*p* value—not significant for all comparisons) as shown in Figure 1C. We further confirmed the presence of CD63 as a typical tetraspanin exosome marker (Figure 1D).

Next, we labeled LFEs with the PKH26 dye and visualized their uptake by small intestinal cells. PKH26 is a lipophilic fluorescent dye widely used for labeling cells for flow cytometry and fluorescence microscopy. The old‐male LFE condition is shown as a representative example; comparable uptake was observed for LFEs from young and old mice of both sexes (YM, OM, YF, and OF) (Figure S1A). We then quantified the proportion of small intestinal epithelial cells labeled with LFEs across all groups and observed no significant differences, consistent with NTA results showing similar vesicle numbers (Figure S1B). Together, these data indicate that downstream functional differences are likely driven by age‐ and sex‐dependent changes in LFE cargo rather than by differences in vesicle abundance, structure, or cellular uptake.

### 3.2 Blood Pressure in Mice

Systolic and diastolic blood pressure were significantly elevated in older mice compared with the young groups (Figure S1A). OM mice had the highest systolic blood pressure (112.38 ± 9.66 mmHg), followed by OF (104.25 ± 6.42 mmHg), with YM (95.51 ± 3.57 mmHg) and YF (92.88 ± 5.84 mmHg) exhibiting lower BP levels (*p* < 0.05, *n* = 8–10/group). Similar findings emerged in relation to diastolic blood pressure: OM: 77.25 ± 2.88 mmHg; OF: 67.32 ± 3.26 mmHg; YM: 63.16 ± 5.78 mmHg; YF: 65 ± 5.21 mmHg (*p* < 0.05, *n* = 8–10/group) (Figure S2A,B). These findings indicate that age is the primary driver of increased blood pressure, with sex modulating the magnitude of this effect, particularly in older animals. This pattern is consistent with age‐associated vascular changes and suggests that older male mice may be more susceptible to hypertensive phenotypes.

### 3.3 Gut Barrier Integrity and Permeability In Vitro

**FIGURE 2:**
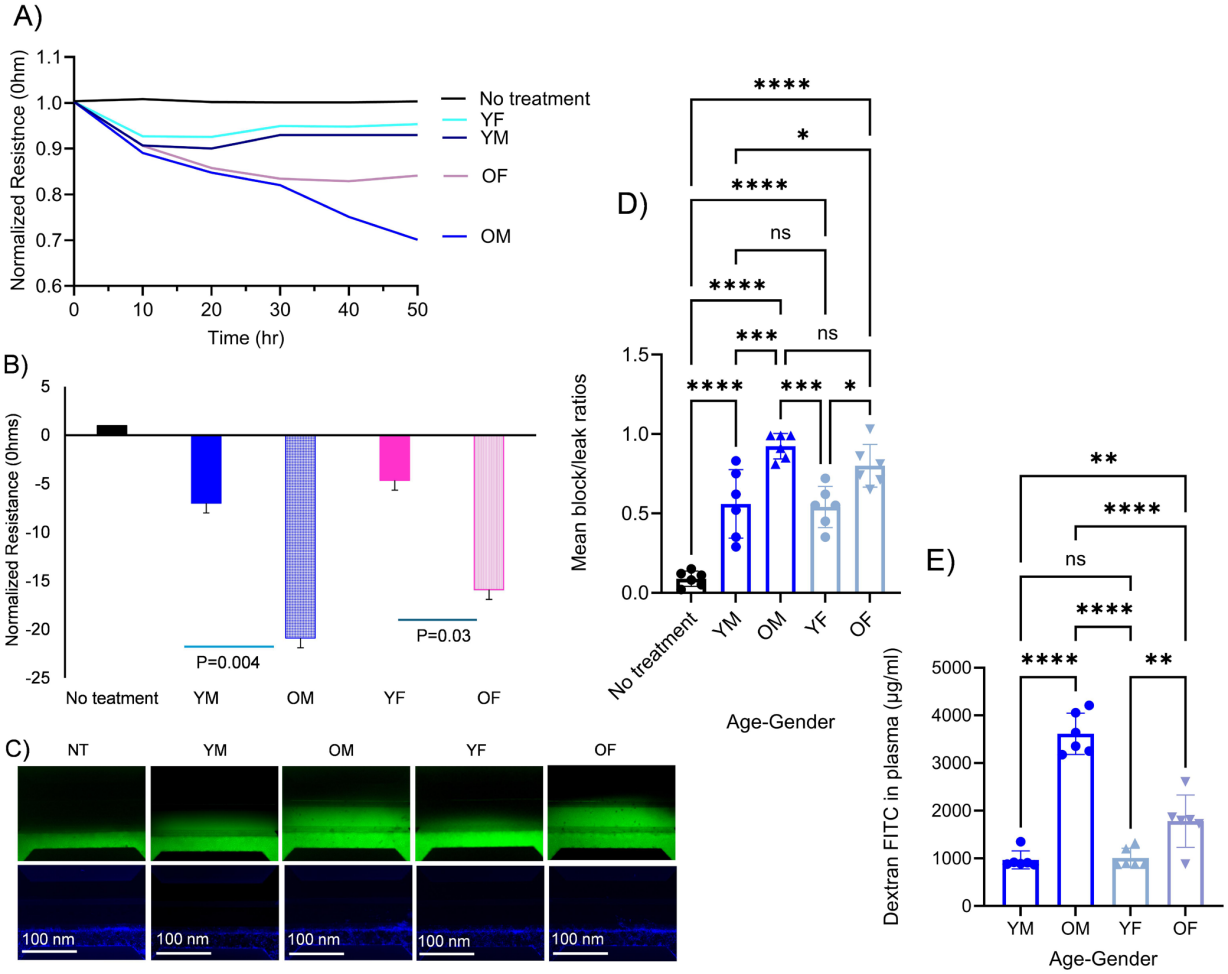
Barrier integrity and permeability in response to luminal fecal exosomes (LFEs) in young and old mice in vitro and in vivo. (A) Electrical resistance was measured by ECIS and normalized to time 0. (B) Mean normalized electrical resistance following LFEs addition. ECIS responses were recorded from old male (OM), old female (OF), young male (YM), and young female (YF) groups using an ECIS Z‐theta system. (C) Representative image of OrganoPlate 3‐lane microfluidic chips perfused with 20‐kDa FITC–dextran. Dextran fluorescence is shown in green, and nuclei stained with DAPI are shown in blue. (D) Quantitative analysis of dextran leakage and barrier permeability in small intestine cells treated with LFEs. Relative fluorescence units (RFUs) were measured in duplicate and averaged. Permeability was expressed as RFUs for the groups being compared. (E) In vivo permeability in response to LFEs derived from young and old mice (male and female) was measured using 4‐kDa fluorescein isothiocyanate (FITC)–dextran. Mice were fasted for 4 h, FITC–dextran was administered by gavage, and plasma was collected 2 h later for fluorescence measurement. Statistical analysis was performed using two‐way ANOVA followed by Tukey's multiple‐comparisons test. Differences were considered significant at *p* < 0.05. * Indicates *p* < 0.01, **p = 0.001, ***p = 0.000, ****p < 0.0001. Sample size was *n* = 6–8 per group.

The intestinal barrier is an important system for communication between the host and the external environment. We therefore examined the effects of LFEs from young and old mice on intestinal barrier integrity (Figure 2). The normalized baseline intestinal barrier resistance (Ohms) for each group was as follows: YM (0.94 ± 0.03), OM (0.84 ± 0.11), YF (0.96 ± 0.02), and OF (0.88 ± 0.06) (Figure 2A). LFEs from YF elicited smaller decreases in resistance compared to YM with similar findings in OF versus OM LFEs (Figure 2B). Actual declines elicited by LFEs from each mouse group were: YM (−7.01 ± −074), OM (−20.88 ± −2.24). YF (−4.65 ± 0.54) and OF (−15.91 ± −1.52). We found significant differences among the following groups: YM versus OM (*p* = 0.005) and OM versus YF versus OF (*p* = 0.03), with no significant differences between OM and OF (*p* = 0.47). These results suggest that both age and sex influence the response of a standard naïve intestinal monolayer barrier, with older males being the most disruptive, as indicated by the marked decline in intestinal cell barrier resistance.

To assess gut permeability, LFEs from young and old male and female mice were applied to small intestinal cells using a microfluidic system. FITC‐dextran was added to the luminal side, and the gut permeability was measured using fluorescence microscopy. As shown in Figure 2C, FITC fluorescence was significantly higher across the intestinal cell layer in OM and OF groups compared to YM and YF groups, indicating that LFEs from older mice increase intestinal cell permeability (Figure 2C,D).

LFEs from older mice impair intestinal barrier integrity and increase gut permeability more than LFEs from young mice. This effect is strongest in older males. Overall, both age and sex influence how fecal‐derived factors disrupt the gut barrier, with aging being the dominant factor.

### 3.4 Gut Barrier Integrity and Permeability In Vivo

Intestinal permeability was assessed in vivo using oral administration of FITC‐dextran fluorescence used as an indicator of gut barrier integrity. As shown in the Figure 2E, significant age‐ and sex‐dependent differences in intestinal permeability were observed. Young male (YM) mice exhibited relatively low plasma FITC‐dextran levels (970.11 ± 170.78), indicating preserved intestinal barrier function. In contrast, old male (OM) mice showed a marked increase in circulating FITC‐dextran (3615.33 ± 396.27) compared with YM mice (970.11 ± 170.78), reflecting significantly increased intestinal permeability (*p* < 0.0001). This finding indicates a pronounced age‐associated impairment of gut barrier integrity in males. Among females, young female (YF) mice displayed plasma FITC‐dextran levels comparable to those of YM mice, with no significant difference between the two young groups (ns). However, old female (OF) mice demonstrated significantly elevated FITC‐dextran levels (1781.34 ± 500.85) compared with YF mice (1012.15 ± 183.76) (*p* < 0.01), indicating an age‐related increase in intestinal permeability in females as well.

Sex‐based differences were also evident. OM mice exhibited significantly higher plasma FITC‐dextran levels (3615.33 ± 396.27) than OF mice (1781.34 ± 500.85) (*p* < 0.01), suggesting a greater degree of gut barrier dysfunction in aging males relative to aging females. No significant sex difference was observed between YM and YF groups. Together, these data demonstrate that intestinal permeability increases with age in both sexes, with a more pronounced disruption observed in old males. These findings in vivo provide strong support for age‐ and sex‐dependent alterations in gut barrier integrity and are consistent with the functional changes observed in vitro. Our data suggest that aging changes the “messages” carried by gut‐derived exosomes, and those messages can make the intestinal barrier more prone to leaking, which could contribute to age‐related inflammation or disease.

### 3.5 GTT and ITT

**FIGURE 3:**
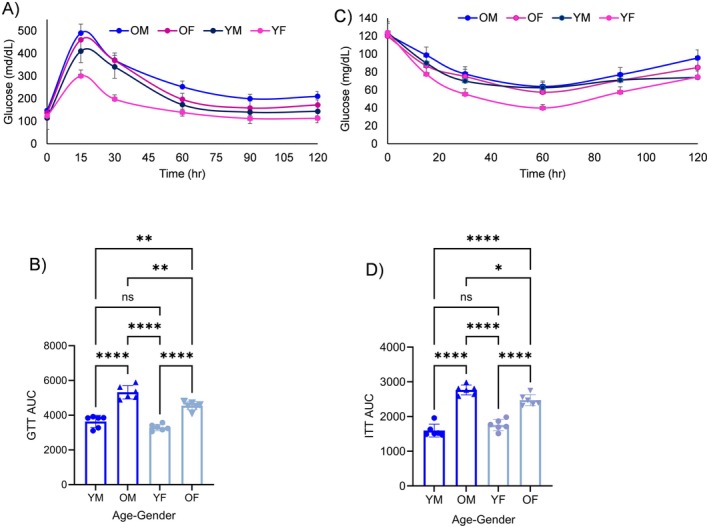
Glucose insulin tolerance test (GTT) and insulin tolerance test (ITT) response in naïve young mice gavage fed with LFEs derived from young and old male and female mice. (A) GTT: Mice were fasted for 4 h, administered oral glucose (2 g/kg), and blood glucose was measured at 0, 15, 30, 60, and 120 min. (B) GTT area under the curve (AUC). (C) ITT: Mice were fasted for 4 h and injected with human insulin (0.75 U/kg); blood glucose was measured at 0, 20, 40, 60, and 120 min. (D) ITT AUC. Data are presented as mean ± SD. Statistical significance was assessed by two‐way ANOVA with repeated measure. * Indicates *p* < 0.01, **p = 0.001, ***p = 0.000, ****p < 0.0001 between groups. Sample size was *n* = 6–8 per group.

To evaluate the effects of LFEs on insulin sensitivity in mice, we conducted glucose tolerance tests (GTT) and insulin tolerance tests (ITT) after administering LFEs derived from young and old mice into young naïve recipient mice. Blood glucose levels were significantly higher in mice receiving LFEs derived from OM and OF mice compared to those receiving LFEs from YM and YF mice (Figure 3A). Area under the curve (AUC) analysis revealed impaired glucose tolerance in old mice LFEs‐treated mice (5322.33 ± 349.59 mg/dL) compared to young mice LFEs‐treated mice (3640.51 ± 320.19 mg/dL, *p* < 0.0001; Figure 3B). These data conclude that LFEs carry age‐dependent factors that negatively affect metabolic regulation. LFEs from old mice are sufficient to induce glucose intolerance in otherwise healthy young mice, suggesting that aging alters circulating extracellular components in a way that promotes insulin resistance.

Insulin injections during ITT experiments resulted in a statistically significant reduction in blood glucose levels at 15 and 30 min (Figure 3C). However, gavage with LFEs from old mice significantly reduced insulin sensitivity compared to LFEs from young mice (Figure 3C). The AUC was significantly higher in OM‐treated mice (2767.67 ± 128.97) compared to young male (YM) LFEs‐treated mice (1597.17 ± 168.69, *p* = 0.0001). Similarly, old female (OF) LFEs‐treated mice exhibited higher AUC values (2472.11 ± 146.54) compared to young female (YF) LFEs‐treated mice (1755.33 ± 146.72, *p* = 0.0001) (Figure 3D). Taken together, LFEs from old mice induce insulin resistance in young recipient mice. Although insulin can still reduce blood glucose, its effectiveness is diminished, indicating that aging‐associated LFEs interfere with insulin signaling or downstream glucose uptake. Our data suggests that aging changes the biological cargo of fecal exosomes, and these altered LFEs can promote glucose intolerance and insulin resistance, potentially contributing to age‐related metabolic diseases.

### 3.6 16S rRNA Sequencing of LFEs From Young and Old Mice

Next, we performed 16S rRNA sequencing on fecal samples used to derive LFEs from old mice (donor) and on fecal samples from recipient mice receiving LFEs from young and old mice (i.e., 4 experimental groups: young with LFEs [YW], young without LFEs [YN], old without LFEs [ON], and old with LFEs [OW]). These comparisons revealed significant differences in microbial abundance and diversity across various taxa (Table S1). Shifts in bacterial community structure were observed, with distinct log_2_ fold changes and statistical significance (Table S1).

In YN versus YW, YN had higher levels of Lachnospiraceae (log_2_ FC = 3.29), Peptococcaceae, Erysipelatoclostridiaceae, and Clostridia_UCG‐014. YW showed higher Bifidobacteriaceae and Candidatus_Saccharimonas. In ON versus YN, ON had increased Defluviitaleaceae, Erysipelatoclostridiaceae, and Lachnospiraceae, while Muribaculaceae and Clostridia_vadinBB60 were more abundant in YN. OW versus ON showed enrichment of Dubosiella, Muribaculaceae, Bifidobacterium, and Faecalibaculum in OW. In OW versus YW, OW had higher Dubosiella, Bifidobacterium, and Peptococcaceae, while Oscillibacter was lower.

The amplicon sequence variants (ASVs) that were detected in each group were: YN (83.17 ± 3.02), YW (91.01 ± 3.83), ON (74.40 ± 3.99), and OW (65.5 ± 5.19). Significant differences were observed between YN versus YW (*p* = 0.03), YN versus ON (*p* = 0.02), YN versus OW (*p* = 0.0001), YW versus OW (*p* = 0.0001), and ON versus OW (*p* = 0.01). These data show that LFEs significantly and age‐dependently reshape the gut microbiota, affecting both community composition and diversity, with distinct outcomes in young versus old recipients. Age alone alters the microbiome, but LFEs further modify this baseline in ways that are not uniform across age groups.

### 3.7 Extracellular Vesicles Cargo

#### 3.7.1 LFEs Proteomics (Mouse and Bacterial Genomes)

**FIGURE 4:**
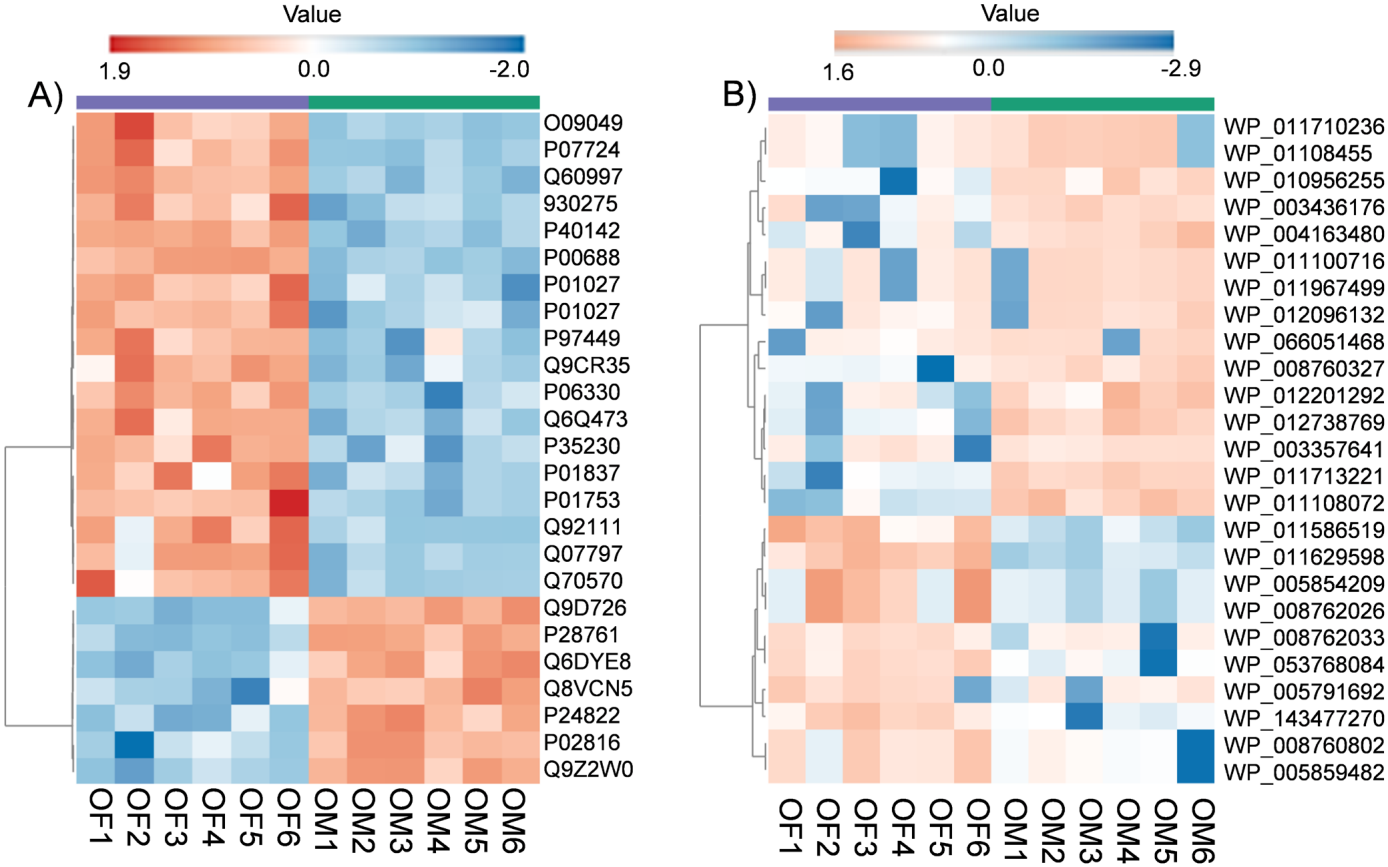
Proteomic profiling of luminal fecal exosomes (LFEs) was performed in old male and female mice using liquid chromatography–mass spectrometry (LC–MS) to characterize host‐derived proteins. Differentially expressed proteins (DEPs) were identified by searching against the mouse genome and bacterial databases. (A) Heatmap analysis shows DEPs for mouse OM versus OF, and (B) bacterial OM versus OF. Red indicates upregulated proteins, and blue indicates downregulated proteins. Significant protein IDs are shown on the right side of each heatmap. Statistical significance was defined as *p* < 0.05 with a fold change > 2.0, *n* = 6 per group.

To define age‐ and sex‐dependent molecular changes in luminal fecal exosome (LFE) cargo, we performed LC–MS/MS proteomic profiling of host‐ and microbe‐derived proteins. Heatmap analysis of differentially expressed proteins (DEPs) revealed clear age‐ and sex‐specific patterns. Across samples, ~500–750 proteins were detected, with 122 shared across groups. In the OM versus OF comparison using the mouse genome, Annexin A2 and Annexin A4 were significantly upregulated in OM, whereas proteins involved in digestion, metabolism, and immunity (including Reg3g, albumin, transketolase, α‐amylase, and Klk1) were downregulated (Table S2). Additional comparisons identified 95 DEPs in OM versus YM, 89 in OF versus YF, 64 in OM versus OF, and 45 in YM versus YF (Tables S3–, S5), respectively. Clustering analyses further demonstrated distinct proteomic trajectories across age and sex groups (Figure S4 and Figure 4). Proteomic profiling of luminal fecal exosomes (LFEs) comparing old male (OM) and old female (OF) mice is presented as heatmaps. Host‐derived mouse protein profiles are shown in Figure 4A, while bacterial protein profiles are shown in Figure 4B.

Functional enrichment analysis of GO and KEGG pathways was performed using both mouse and bacterial proteomic datasets. In OM versus OF, pathways such as Pancreatic Secretion, Renin–Angiotensin System, and Carbon Metabolism highlighted differences in nutrient processing and cardiometabolic function. In OM versus YM, comparisons revealed enrichment of Proteasome, Pancreatic Secretion, and neurodegeneration‐related pathways (Parkinson's, Alzheimer's, ALS), alongside shifts in digestion and absorption. In OF versus YF, Proteasome, Pancreatic Secretion, and neurodegenerative pathways (Alzheimer's, prion disease) were enriched, reflecting aging‐related vulnerability. YM versus YF comparisons showed sex‐specific differences in Pancreatic Secretion, Protein and Fat Digestion, and Glycerolipid Metabolism, indicating distinct metabolic traits in young mice (Tables S7–, S9).

Bacterial EV proteomics revealed marked age‐ and sex‐dependent differences. In OM versus OF, several ribosomal proteins, including 30S S5 and 50S L7/L12 from Bacteroides and related taxa, were reduced in OM, suggesting decreased translational activity. Shifts in elongation factors, metabolic enzymes, and energy‐transfer proteins (e.g., phosphopyruvate hydratase, NADP‐specific glutamate dehydrogenase, electron transfer flavoprotein, UgpC) indicated altered microbial metabolism and energy utilization. Reduced flagellin suggested decreased motility, while changes in enzymes involved in amino acid and carbohydrate metabolism reflected functional remodeling.

OM versus YM analysis showed increased abundance of microbial proteins linked to translation, metabolism, and structural functions in OM. In OF versus YF, aging was associated with enrichment of proteins involved in protein synthesis and diverse metabolic pathways, including elongation factor Tu, malate dehydrogenase, formate‐tetrahydrofolate ligase, acyl‐CoA carboxylase, and electron transfer flavoprotein. In YM versus YF, only modest differences were observed, including mild upregulation of ketol‐acid reductoisomerase, suggesting subtle sex‐related metabolic variation (Tables S10–, S14). Together, integrated host–microbial LFE proteomics reveals pronounced age‐ and sex‐specific functional remodeling, providing mechanistic insights into gut‐derived molecular changes beyond taxonomic profiling.

### 3.8 LFEs Small miRNA Sequencing and Network

**FIGURE 5:**
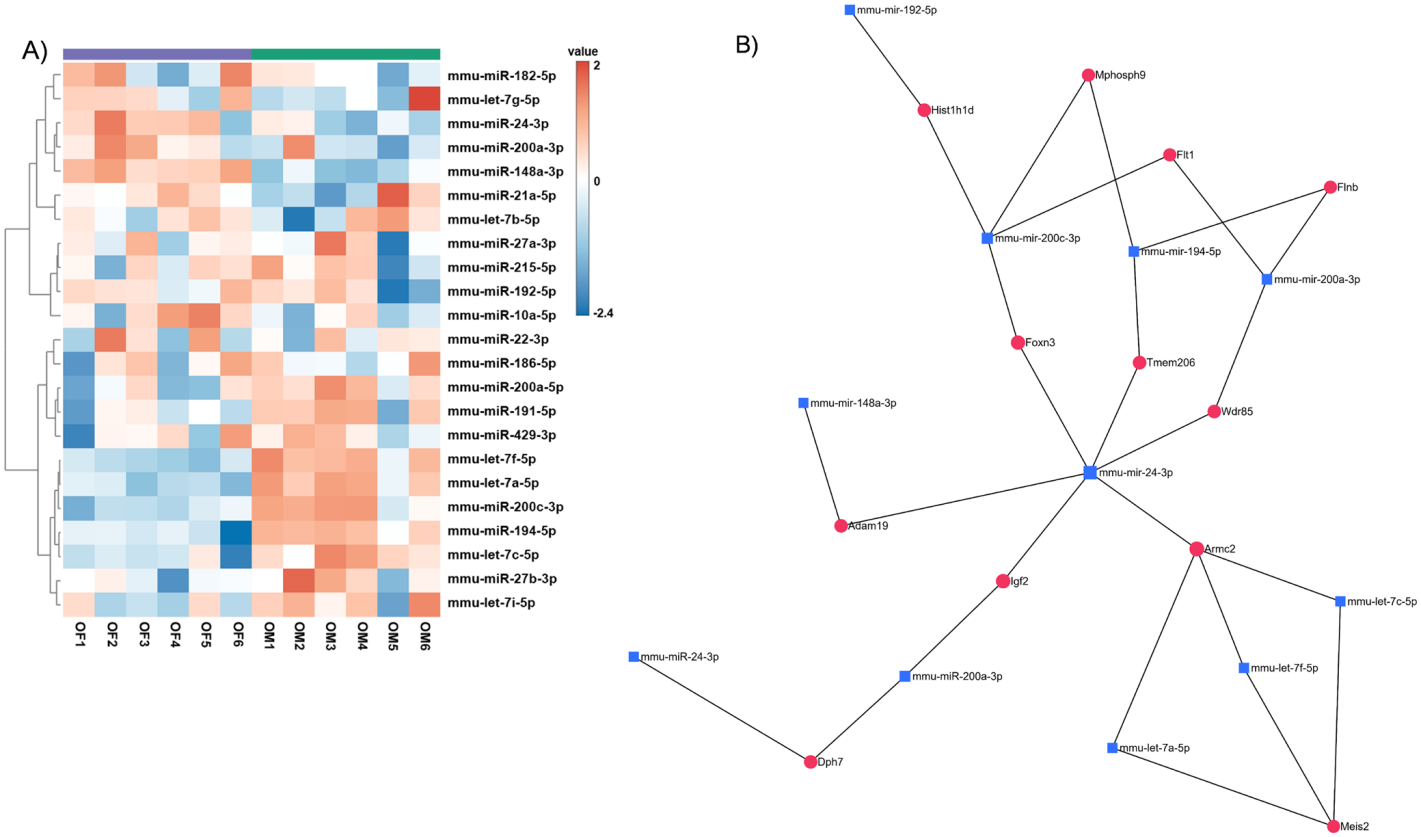
Heatmap and network analysis of luminal fecal exosome (LFE) miRNAs in aged mice. (A) Heatmap of differentially expressed miRNAs comparing old male (OM) and old female (OF) mice. Red indicates upregulation and blue indicates downregulation. Significantly associated protein IDs are shown to the right of the heatmap. (B) miRNA–mRNA interaction network for the OM versus OF comparison. miRNAs are shown as blue nodes, and their corresponding minimum gene targets are shown as red nodes. Edges represent predicted interactions, highlighting miRNA regulatory relationships between groups. Statistical significance was defined as *p* < 0.05 with a fold change > 2.0. *n* = 6 per group. *n* = 6/group.

miRNAs are key functional components of luminal fecal exosome (LFE) cargo and play an important role in intercellular communication and downstream biological regulation effects (Sanz‐Ros et al. 2025). To identify age‐ and sex‐associated differences in miRNA expression, we performed small RNA sequencing on LFEs isolated from young and old male and female mice. In old males versus old females (OM vs. OF), we identified 11 differentially expressed miRNAs (DEMs) (Table S14). We also identified five DEMs in OM versus young males (YM), five in OF versus young females (YF), and two in YM versus YF (Table S14). Differentially expressed miRNAs (DEMs) were visualized using heatmaps. The OM versus OF comparison is shown in Figure 5A, while OM versus YM, OF versus YF, and YM versus YF are shown in Figure S5A–C, respectively. The significant miRNAs identified between groups were further used to construct miRNA–gene interaction networks. The networks for the 11 significant miRNAs in the OM versus OF comparison (Table S14) are shown in Figure 5B, while the networks for OM versus YM, OF versus YF, and YM versus YF are shown in Figure S6A–C, respectively. Overall, LFE miRNA profiling revealed distinct age‐ and sex‐dependent expression patterns. Notably, these differences were apparent even in young mice, suggesting early establishment of sex‐specific miRNA regulation that may contribute to divergent physiological functions and aging trajectories.

### 3.9 miRNAs Target Predications Genes and KEGG Pathway

Putative miRNA target genes were subjected to KEGG pathway enrichment analysis to investigate molecular processes associated with age‐ and sex‐dependent miRNA expression changes (Table S15). In OM versus OF, enriched pathways included axon guidance, MAPK, and PI3K–Akt signaling, reflecting sex‐specific differences in neural and intracellular signaling. Cancer‐related pathways, mTOR and ErbB signaling, and autophagy/mitophagy pathways were also prominent. Additional enrichment of Rap1, Ras, and TGF‐β signaling suggests altered cell communication and immune‐related mechanisms. In OM versus YM, enriched pathways included Polycomb Repressive Complex and axon guidance, indicating altered gene regulation and neural processes with aging. Key signaling pathways such as MAPK and mTOR were significantly enriched, along with autophagy‐related pathways (autophagy and mitophagy), highlighting changes in cellular growth, stress response, and quality control. Cancer‐related pathways, longevity‐regulating pathways, and cell adhesion pathways (adherens junction and Rap1 signaling) were also enriched, suggesting broader impacts on aging‐associated disease and cell communication. In OF versus YF, target genes were strongly enriched for PI3K–Akt signaling and focal adhesion, pathways central to cell survival and proliferation. Multiple cancer‐related pathways were significant, along with longevity‐regulating and metabolic pathways, including insulin resistance and choline metabolism. Enrichment of apoptosis, autophagy, and MAPK/mTOR signaling further supports roles for these miRNAs in regulating cell death, metabolism, and growth during aging. No significant pathways were identified in YM versus YF.

The enriched pathways point to broad changes in epithelial cell regulation, structure, and stress responses. Key growth and survival pathways (MAPK, PI3K‐Akt, mTOR, Ras, p53, FoxO) suggest altered control of cell turnover, metabolism, and damage responses, consistent with aging‐related shifts and impaired repair. Pathways involved in cytoskeletal regulation, focal adhesion, and cell–cell junctions indicate weakened epithelial architecture, supporting evidence of reduced gut barrier integrity and increased permeability. Changes in autophagy and mitophagy highlight disrupted cellular maintenance and mitochondrial quality control, processes commonly affected by aging and inflammation. Enrichment of Polycomb‐related pathways suggests more stable, long‐term changes in gene regulation rather than only transient signaling effects. Neural‐associated pathways such as axon guidance and neurotrophin signaling likely reflect roles in cell migration, polarity, and tissue organization rather than neuronal activity. Cancer‐related pathways appear because they share core signaling networks involved in growth, survival, adhesion, and inflammation, not because of cancer itself. Overall, these findings indicate that the condition studied influences epithelial stability, intracellular signaling, stress handling, and long‐term regulatory programs in ways that align with observed gut barrier dysfunction.

**FIGURE 6:**
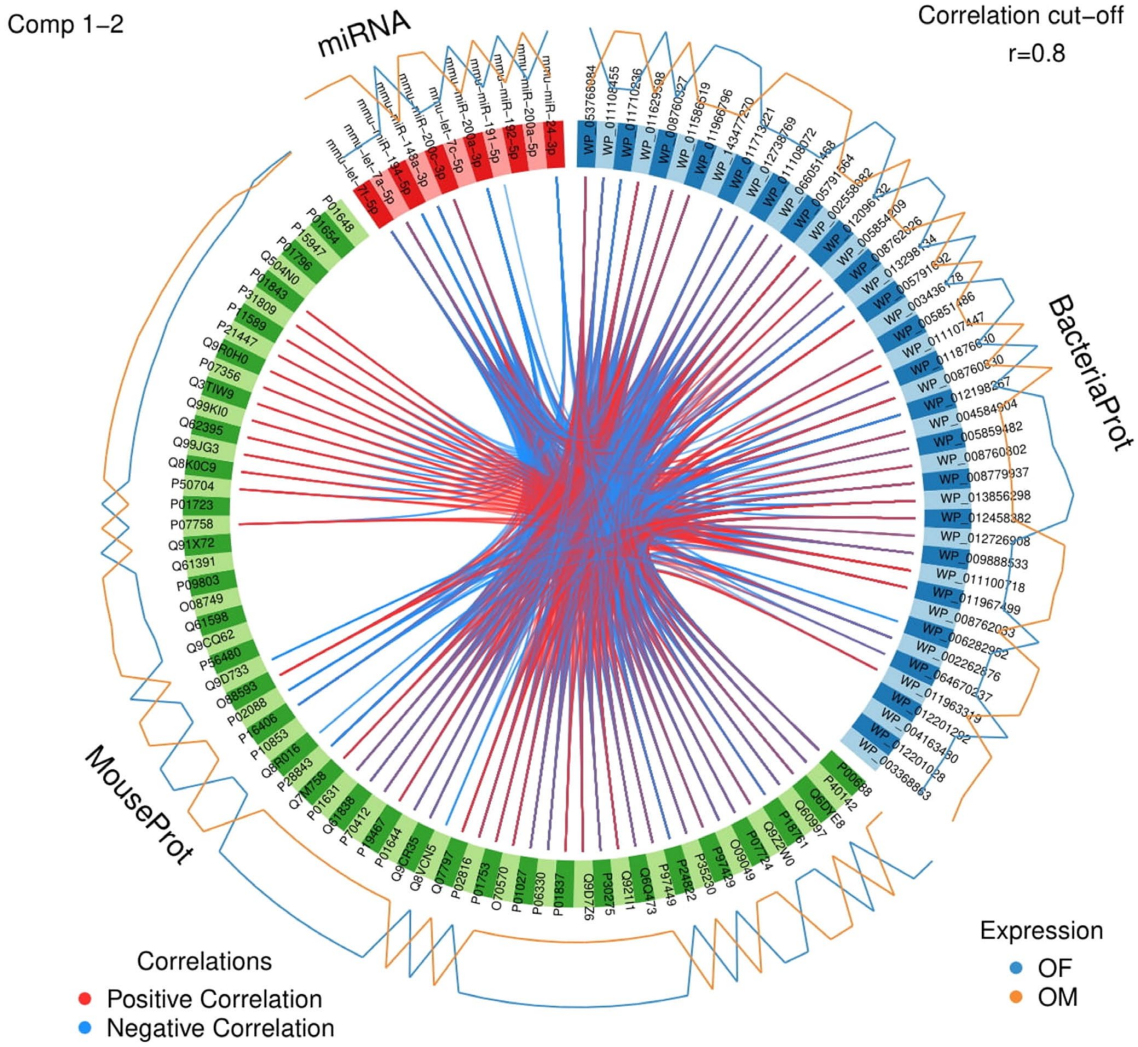
Circular plot integrating miRNAs, bacterial genomes, and mouse proteins. Outer rings represent different data categories: MiRNAs (blue), bacterial genomes (green), and mouse proteins (yellow). Circular plot comparing old male (OM) and old female (OF) mice. Lines within the circle indicate significant interactions or functional associations identified through bioinformatic analysis. Red lines represent positive correlations, and blue lines represent negative correlations. Only correlations meeting the cutoff of |*r|* ≥ 0.8 are shown. These connections highlight potential relationships among miRNAs, mouse proteins, and bacterial proteins.

To further define miRNA–gene regulatory relationships, miRNet was used to construct miRNA–target interaction networks (Figure 5B). In OM versus OF, 11 miRNAs were associated with predicted gene targets forming an interaction network (Figure 6B). The OM versus YM comparison identified five miRNAs connected through a shared gene network (Figure S6A). In OF versus YF, five miRNAs were detected, with only two (miR‐192‐5p and miR‐429‐3p) sharing a common target gene, Hist1h1d (Figure S6B). In the YM versus YF comparison, two miRNAs were identified; however, no shared gene interactions were observed, as each miRNA targeted distinct genes (Figure S6C). The target networks for miRNA‐151‐5p and miRNA‐191‐5p are shown in Figure S6C1,C2, respectively.

### 3.10 Integration of LFEs Proteomics and Small miRNAs

Using multi‐block analysis, circular plots were generated to integrate data from miRNAs, host‐derived proteomics, and bacteria‐derived proteomics. The outer ring of each plot is divided into three sections: miRNAs, mouse proteins, and bacterial proteins. Lines within the circle indicate correlations (*r* ≥ 0.8 as a threshold), with red lines representing positive correlations and blue lines representing negative correlations, visualizing the interactions among these biomolecules. The internal lines reflect the correlation types, while the outer lines indicate expression levels across two groups. The plots highlight age‐related expression differences between older mice (OM vs. OF), denoted by blue and orange lines, respectively (Figure S6). Specific circular plots compare OM versus YM (Figure S7A), OF versus YF (Figure S8), and YM versus YF (Figure S9). Collectively, these integrative analyses indicate that LFEs carry coordinated molecular signatures linking bacterial proteins, host proteins, and miRNAs in an age‐ and sex‐dependent manner. The strong correlation patterns observed among these biomolecules suggest that LFEs serve as a platform for microbiome–host communication that changes with aging. Comparisons between young and old mice reveal age‐associated remodeling of these interaction networks, while differences between males and females highlight sex‐specific regulatory pathways that are particularly evident in young animals. Together, these data support the conclusion that aging and sex shape distinct LFE‐mediated molecular networks through which the microbiome may influence host physiology.

## 4 Discussion

In this study, we isolated and characterized luminal fecal exosomes (LFEs) derived from young and old mice and assessed their effects on gut barrier integrity and host metabolism. We show that LFEs from old mice induce deterioration of gut barrier function both in vivo and in vitro, resulting in increased intestinal permeability compared with LFEs from young mice. In addition, LFEs from old mice impaired insulin sensitivity and glycemic control in naïve recipients. These findings demonstrate that age‐associated differences in LFEs are sufficient to induce measurable functional changes in gut barrier integrity and metabolic outcomes. We further observed that LFEs from young and old mice differentially altered gut microbial composition in recipient animals, as assessed by 16S rRNA sequencing. These findings are consistent with the concept that host–microbe interactions in the gut lumen occur within a competitive ecosystem, in which host‐derived factors influence microbial population dynamics (Chang and Kao 2019; Sarkar et al. 2024). Proteomic and miRNA profiling of LFEs revealed distinct age‐ and sex‐associated differences in cargo derived from both host and microbial sources (Table S14). These analyses identified numerous differentially expressed proteins and miRNAs mapping to pathways involved in immune regulation, metabolism, protein homeostasis, and cellular stress responses. Given the exploratory nature of these multi‐omics analyses, pathway enrichment results should be interpreted as hypothesis‐generating rather than definitive evidence of pathway‐level dysregulation. Nonetheless, the observed signatures align with known biological processes implicated in normal aging and in age‐related disease (Hou et al. 2022; Li et al. 2021; Sun et al. 2023).

Aging is associated with well‐established physiological changes, including chronic inflammation, impaired nutrient sensing, mitochondrial dysfunction, and dysbiosis (Lopez‐Otin et al. 2013). Microbiome studies in older adults broadly describe age‐related shifts in gut microbial composition as well as alterations linked to aging‐associated disorders (Campisi et al. 2019; Ghosh et al. 2022). In this context, our findings extend on prior work by identifying LFEs as a functional component of fecal material capable of transmitting age‐associated effects on gut barrier integrity and metabolic regulation (Figure 2). In healthy individuals, the intestinal barrier plays a central role in maintaining gut homeostasis by regulating interactions among microbiota, epithelial cells, mucus layers, and immune cells (Pham et al. 2018; Sayoc‐Becerra et al. 2020). Disruption of this barrier inevitably results in increased permeability and altered host–microbe signaling (Di Vincenzo et al. 2024; Pham et al. 2018).

Extracellular vesicles derived from intestinal microbes have been proposed as mediators of interspecies and interorgan communication (Cuesta et al. 2021), with evidence that gut‐derived EVs can enter the circulation and influence peripheral tissues (Xie et al. 2025). In the present study, we establish age‐ and sex‐related differences in LFEs cargo and demonstrate that LFEs from aged donors alter gut microbiome composition and gut barrier function in naïve recipients. Notably, LFEs from old mice induced significant shifts in taxa such as Muribaculaceae and Bifidobacterium, which have previously been linked to aging‐related changes in gut health (Bradley and Haran 2024). Our findings highlight age‐ and genotype‐related shifts in the gut microbiome. In older mice, increased levels of bacteria like *Dubosiella* and *Bifidobacterium* may reflect adaptive responses to aging, while declines in *Muribaculaceae* and *Christensenellaceae* could signal weakening gut function and immunity. These microbial changes may drive systemic and intestinal inflammation, contributing to age‐related diseases. Targeted interventions—such as diet or probiotics—could help counteract this decline.

### 4.1 Luminal Fecal Exosomes Cargos

Multi‐omics data and advanced bioinformatics are essential for understanding the biological and biomedical features of LFEs in both aging and young animals. EVs act as mediators by carrying diverse biomolecules, making multi‐omics approaches critical for defining their functions. As EVs reflect their cells or tissues of origin, they support local communication, help identify their sources, and influence distant cells and microenvironments. For example, EV proteomics often identifies tissue‐specific proteins, making EVs valuable for monitoring pre‐disease and disease states in patient blood (Muraoka et al. 2022; Sultan et al. 2021). Integrative multi‐omics analyses can further illuminate EV functions. Multi‐omics approaches have been used in aging (Liu, Lu, et al. 2024; Wang et al. 2024; Wei et al. 2024), but studies using gut microbiota EVs in aging are scarce. The current experiments provide insights into the multi‐omic changes of LFEs in aging and pave directions into further research opportunities.

### 4.2 Proteomics

Most gut microbiome studies focus on taxonomic and functional profiles using 16S rRNA and metagenomics. In contrast, we analyzed LFEs cargo proteomics to examine gut microbiota and host–microbiome interactions in young and old mice (Tables S2 and S13). We identified pathways linked to gut homeostasis and aging. Immune‐related proteins, including Jchain, were downregulated in old mice (OM), indicating age‐associated immune decline. Proteasome subunits (Psma3, Psmb4) were moderately upregulated, suggesting compensatory changes in protein degradation. Digestive and metabolic enzymes such as Klk1 and Prss2 were downregulated in OM, consistent with age‐related metabolic alterations. Klk1 is involved in inflammation control, oxidative stress, and tissue repair, processes closely tied to aging and age‐related diseases (Nokkari et al. 2018). Prss2 plays a role in proteolysis and tissue remodeling and has been linked to age‐associated pancreatic disorders (Qin et al. 2023; Whitcomb 2013). Increased histone H4 levels in OM suggest epigenetic changes with aging. Overall, these findings indicate reduced immune and digestive function alongside adaptive increases in proteasome activity, reflecting altered protein turnover and energy demands in older mice.

We also examined pathway enrichment across age and sex groups. KEGG analysis showed significant enrichment of proteasome pathways in older mice (OM, OF) compared to younger groups (YM, YF), consistent with age‐related disruptions in protein quality control (Hegde et al. 2023; Stolzing and Grune 2001). Pancreatic Secretion pathways were enriched in OM, supporting known declines in digestive enzyme output with age (Lohr et al. 2018). Neurodegeneration‐related pathways, including Parkinson's, Alzheimer's disease, and Spinocerebellar Ataxia, were also enriched, reflecting increased susceptibility to age‐related neurological disorders (Zhang et al. 2023). Metabolic pathway enrichment, supported by insulin and glucose tolerance tests, pointed to altered energy metabolism and insulin sensitivity in aging (Palmer and Jensen 2022). Enrichment of the Renin–Angiotensin System suggests age‐related cardiovascular changes linked to hypertension and heart disease (Ahmad et al. 2023).

Finally, we identified age‐ and sex‐associated proteins involved in KEGG pathways related to protein digestion and absorption (MEP1A, CPA1, CTRB1, PRSS2, MEP1B, CELA2A, MME) and Alzheimer's disease (multiple proteasome subunits and MME), particularly in OM versus YM. Several proteins, including MME, were shared between digestive and neurodegenerative pathways, suggesting overlap between gut dysfunction and brain health. These findings support the idea that age‐related gut metabolic stress may contribute to systemic inflammation and exacerbate neurodegenerative processes.

### 4.3 Small miRNAs

Extracellular vesicles (EVs) carry miRNAs that regulate gene expression and can be transferred between cells, thereby influencing biological functions and disease processes (Khalyfa et al. 2016; Liu, Park, et al. 2024). Pathway enrichment analyses across multiple comparisons (OF vs. YF, OM vs. OF, OM vs. YM, YM vs. YF) revealed distinct age‐ and sex‐specific signaling pathways (Figure 5, Figure S5). Overall, miRNA expression profiles clearly differed by age and sex. Several miRNAs, including miR‐194‐5p, let‐7f‐5p, and mmu‐miR‐26a‐5p, were upregulated in older mice, while others, such as let‐7c‐5p and miR‐24‐3p, were downregulated in both older males and females.

In OM versus YM comparisons, enrichment of growth and metabolic pathways, including Polycomb repressive complex, axon guidance, mTOR, and MAPK signaling, indicates pronounced aging‐related changes in males. Pathways linked to stem cell pluripotency, mitophagy, and autophagy suggest reduced cellular repair capacity with age. These pathways regulate epigenetic control, neuronal connectivity, growth signaling, and stress responses. Notably, age‐related disruption of mTOR and MAPK signaling is associated with altered metabolism, senescence, and impaired autophagy, contributing to stem cell exhaustion and reduced regenerative potential (Chen et al. 2021). Enrichment of cancer‐related pathways, such as ErbB signaling and proteoglycans in cancer, points to increased tumorigenic risk in aging males through dysregulated growth signaling and altered extracellular matrix interactions (Baxter 2023).

In OF versus YF comparisons, pathways related to cell growth, cancer, and longevity were prominently enriched. The PI3K–Akt pathway, a key regulator of cell survival, metabolism, and proliferation, was strongly represented and is commonly dysregulated in age‐related cancers, including colorectal and breast cancer (Rascio et al. 2021; Zaryouh et al. 2022). Enrichment of FoxO signaling and stem cell regulation pathways highlights roles in longevity, oxidative stress control, and stem cell maintenance, processes that decline with age and increase disease susceptibility. Pathways involved in cell adhesion and growth factor signaling, such as focal adhesion and EGFR, were also elevated, reflecting age‐associated changes in cellular growth dynamics (Zaryouh et al. 2022).

In OM versus OF comparisons, enrichment of axon guidance, PI3K–Akt, and MAPK signaling pointed to sex‐specific differences in growth and survival pathways. Additional enrichment of ErbB and mTOR signaling underscores altered growth factor and metabolic regulation in aging males (Bjedov and Rallis 2020; Huang et al. 2017; Zhu et al. 2024). Overall, these patterns highlight distinct age‐ and sex‐dependent biological processes.

### 4.4 Integrating Proteomics and miRNAs

We next examined how LFEs differentially expressed proteins, mapped to mouse and bacterial genomes or miRNAs, integrate across the four experimental groups. In OM versus YM, four of five miRNAs (mmu‐miR‐194‐5p, mmu‐let‐7f‐5p, mmu‐miR‐26a‐5p, mmu‐miR‐200b‐3p, mmu‐miR‐24‐3p) showed positive correlations with proteomic changes. In OF versus YF, all five miRNAs (mmu‐miR‐192‐5p, mmu‐let‐7c‐5p, mmu‐miR‐29a‐3p, mmu‐miR‐148b‐3p, mmu‐miR‐429‐3p) were significantly correlated with proteomic data. Similar but weaker associations were observed in OM versus OF and YM versus YF comparisons.

To assess potential functional implications, we investigated how LFEs influence microbiota composition, diversity, and gut homeostasis in an age‐ and sex‐dependent manner. Exosomes from young mice were transplanted into old mice, followed by multi‐omics profiling of exosomal cargo. In vitro experiments further showed that LFEs modulate gut barrier integrity and metabolic function. Our findings point to LFEs as key mediators of gut and systemic communication, with growing evidence pointing to their role in aging. In aged male and female mice, these vesicles may impact health span by carrying microbial signals that influence inflammation, metabolism, and immune responses.

The major novelty of this study includes our demonstration that LFEs differ significantly by age and sex, and that LFEs derived from aged mice are sufficient to impair gut barrier integrity and alter metabolic outcomes in naïve recipients. These findings support a role for LFEs as functional mediators of age‐associated changes in gut physiology and host–microbiota interactions.

We would like to reiterate that the novelty of our study lies in showing that gut luminal exosomes retain biological activity that can collectively induce senescence‐associated phenotypes or, conversely, alleviate them. This complements prior reports showing that either plasma factors or the gut microbiota can drive similar phenotypic changes, but it identifies luminal exosomes as an additional, previously underappreciated contributor.

Several limitations should also be mentioned. Because of restricted sample availability, qPCR, Western blotting, or functional assays to validate individual hits were not possible. Nonetheless, several top candidate miRNAs and proteins identified through our omics analyses—particularly those involved in tight junction regulation, immune signaling, and metabolic control—are being prioritized for targeted validation in ongoing and future studies. Ultracentrifugation is the most widely used method for extracellular vesicle (EV) isolation and is often considered the gold standard; however, when applied to fecal samples, it yields EV‐enriched preparations rather than pure EVs. Given the high biological complexity of fecal matter, ultracentrifugation can co‐isolate bacterial outer membrane vesicles, microbial and host cell debris, undigested dietary particles, protein–lipid aggregates, non‐vesicular extracellular nanoparticles, and other macromolecular complexes. In addition, extracellular enzymes and nucleic acid–protein complexes may sediment under high centrifugal forces or associate with vesicle surfaces. This ultracentrifugation technique is not applicational to large numbers of human samples which is ultimately the translational goal of this work. Metabolites and lipopolysaccharides can also co‐precipitate during LFEs isolation. High‐speed centrifugation can also damage EV membranes, and because the method relies on size and density, non‐exosomal particles may co‐precipitate during isolation. These potential contaminants may influence particle counts, molecular cargo, and observe biological effects; therefore, the results should be interpreted with appropriate caution. Nevertheless, these limitations are common to current EV isolation approaches, as there is no universally accepted standard method. To mitigate these concerns, positive and negative EV markers were used to assess sample purity. Because the primary focus of this study was the multi‐omics characterization of LFE cargo, including proteins and miRNAs, metabolomic profiling was not performed.

We agree that defining the underlying effector pathways is an important next step. Based on our initial multi‐omic analyses, we anticipate that these effects involve multiple interacting causal agents. Dissecting the critical components and demonstrating their specific roles will require dedicated mechanistic studies and is beyond the scope of the current manuscript. Despite the novelty of our findings identifying a role for gut luminal exosomes in aging‐associated phenotypic expression, the effector pathways underlying these effects remain to be delineated.

## 5 Conclusion

Our findings support a mechanistic framework in which aging, in both males and females, reshapes the intestinal microbiota, leading to altered microbe–host communication mediated by LFEs. Using young and aged male and female mice, we propose that age‐associated shifts in microbial composition modify the molecular cargo of LFEs, thereby changing their signaling capacity and downstream effects on gut barrier integrity and systemic metabolic function. Rather than passive byproducts, LFEs appear to function as active signaling intermediates that integrate microbial cues with host physiological responses across the lifespan.

The age‐dependent, sex‐specific differences observed in LFE composition and function indicate that distinct communication networks operate in males and females and may contribute to divergent aging trajectories. We hypothesize that these differences arise from interactions among microbial metabolism, host hormone signaling, and exosome biogenesis pathways, which are differentially regulated with age in each sex. Collectively, these findings position LFEs as a mechanistic link between age‐related microbiota remodeling and metabolic decline in both males and females. Targeting LFE‐mediated signaling pathways may therefore represent a viable strategy to mitigate age‐associated dysfunction and promote healthier, sex‐informed aging.

## Author Contributions

Abdelnaby Khalyfa initiated the project, performed all experiments, analyzed data and drafted the initial version of the manuscript. Lyu Zhen and Trupti Joshi conducted all bioinformatic analyses and reviewed the manuscript. David Gozal participated in the initial phases of the project, provided critical input throughout the experiments, and critically reviewed the manuscript versions. All authors have read and approved the final version of the manuscript.

## Funding

The authors have nothing to report.

## Conflicts of Interest

The authors declare no conflicts of interest.

## Supporting information


**Figure S1:** Uptake of luminal fecal exosomes (LFEs) labeled with PKH26 by naïve mouse small intestine cells in vitro. LFEs were labeled with the PKH26 red, fluorescent cell linker for 24 h. Cells were washed and nuclei were stained with DAPI (blue). As a control, PKH26 was added in the absence of LFEs. (A) Fluorescence images of small intestine cells incubated with PKH26‐labeled LFEs (red). Nuclei are shown in blue (DAPI). (B) Semi‐quantitative of LFEs uptakes images. Scale bar, 100 nm. NS indicates no significant. *n* = 6–8 per group.

**Figure S2:** Blood pressure in young and old Mice. Blood pressures were measured across four groups: young males (YM), young females (YF), old males (OM), and old females (OF). (A) systolic blood pressure, and (B) diastolic blood pressure. Statistical comparisons were made using a two‐way ANOVA to assess the effects of age and sex on these parameters. Significant differences in blood pressure were observed between the young and old groups, indicating age‐related physiological changes. Two‐way ANOVA using Tukey's multiple comparisons test. * Indicates *p* = 0.01, ** *p* = 0.001, *** *p* = 0.0001. *n* = 6–8 per group.

**Figure S3:** Proteomic profiling of luminal fecal exosomes (LFEs) was performed to characterize host‐derived proteins in young and old mice using liquid chromatography–mass spectrometry (LC–MS). Differentially expressed proteins (DEPs) were identified by searching against the mouse genome database. Heatmap analysis illustrates DEPs for (A) OM versus YM, (B) OF versus YF, and (C) YM versus YF. Red indicates up‐regulated proteins, while blue indicates down‐regulated proteins. Significant protein IDs are shown on the right side of each heatmap. Statistical significance was defined as *p* < 0.05 with a fold change > 2.0, *n* = 6 per group.

**Figure S4:** Proteomic profiling of luminal fecal exosome (LFEs) was performed using Liquid Chromatography Mass Spectrometry (LC–MS). Differentially expressed proteins (DEPs) were identified using the search for bacterial genome (microbiota). Heatmap analysis of differentially expressed proteins (DEPs) analysis for (A) OM versus YM, (B) OF versus YF, and (C) YM versus YF. The red color in the heatmap indicates up‐regulation, and the blue color indicates down‐regulation. Significant protein IDs are shown on the right side of each heatmap. Statistical significance was defined as *p* < 0.05 with a fold change > 2.0, *n* = 6 per group.

**Figure S5:** Heatmap of luminal fecal exosomes (LFEs) miRNAs analysis. Heatmap analysis of differentially expressed miRNAs analysis for OM versus YM (A), OF versus YF (B), and YM versus YF (C). The red color in the heatmap indicates up‐regulation, and the blue color indicates down‐regulation. Significant protein IDs are shown on the right side of each heatmap. Statistical significance was defined as *p* < 0.05 with a fold change > 2.0, *n* = 6 per group.

**Figure S6:** Network analysis of significantly differentially expressed miRNAs across group conditions. (A) miRNA‐mRNA interaction network for OM versus YM shows the minimal target genes for the miRNAs specific to this comparison. (B) Network for OF versus YF highlights the differential interactions in older females compared to younger females. (C) Interaction network for YM versus YF depicts miRNA targets differing between young males and young females. Panel C1 for miRNA‐151‐5p and panel C2 for miRNA 191‐5p. Nodes representing miRNAs are shown in blue, while their corresponding minimum gene targets are shown in red color. Edges signify interactions, and the network structure emphasizes miRNA regulatory roles in different group conditions.

**Figure S7:** Circle plot analysis integrating miRNA, bacterial genomes, and mouse genome proteins. Outer rings represent distinct data categories: miRNAs (blue), bacterial genomes (green), and mouse genome proteins (yellow). Circle plots between OM versus YM. Edges connecting nodes signify interactions or functional associations derived from bioinformatics analysis. Red color indicates positive correlation, while blue is negative correlations. Lines inside the circle connect pairs of entities with significant correlations (correlation cut‐off at *r* = 0.8*r* = 0.8*r* = 0.8). These connections indicate potential interactions or dependencies between miRNAs, mouse proteins, and bacterial proteins.

**Figure S8:** Circle plot analysis integrating miRNA, bacterial genomes, and mouse genome proteins. Outer rings represent distinct data categories: miRNAs (blue), bacterial genomes (green), and mouse genome proteins (yellow). Circle plots between OF versus YF. Edges connecting nodes signify interactions or functional associations derived from bioinformatics analysis. Red color indicates positive correlation, while blue is negative correlations. Lines inside the circle connect pairs of entities with significant correlations (correlation cut‐off at *r* = 0.8r = 0.8r = 0.8). These connections indicate potential interactions or dependencies between miRNAs, mouse proteins, and bacterial proteins.

**Figure S9:** Circle plot analysis integrating miRNA, bacterial genomes, and mouse genome proteins. Outer rings represent distinct data categories: miRNAs (blue), bacterial genomes (green), and mouse genome proteins (yellow). Circle plots between YF versus YM. Edges connecting nodes signify interactions or functional associations derived from bioinformatics analysis. Red color indicates positive correlation, while blue is negative correlations. Lines inside the circle connect pairs of entities with significant correlations (correlation cut‐off at *r* = 0.8r = 0.8r = 0.8). These connections indicate potential interactions or dependencies between miRNAs, mouse proteins, and bacterial proteins.

**Table S1:** Alterations in fecal bacterial composition in naïve mice gavage with LFEs derived from young or old mice and their controls without LEFs.

**Table S2:** List of statistically significant differentially expressed luminal fecal exosome (LFE) proteins identified in mouse genome of aged old male (OM) versus old female (OF) Mice.

**Table S3:** List of statistically significant differentially expressed luminal fecal exosome (LFE) proteins identified in mouse genome of aged (OM) versus young (YM) Mice.

**Table S4:** List of statistically significant differentially expressed luminal fecal exosome (LFE) proteins identified in mouse genome of aged (OF) versus young (YF) Mice.

**Table S5:** List of statistically significant differentially expressed luminal fecal exosome (LFE) proteins identified in mouse genome of young male (YM) versus young female (YF) Mice.

**Table S6:** List of statistically significant Gene Ontology (GO) terms, KEGG pathways, and tissue‐specific features identified in mouse genome of aged OM versus OF mice.

**Table S7:** List of statistically significant Gene Ontology (GO) terms, KEGG pathways, and tissue‐specific features identified in mouse genome of aged OM versus YM mice.

**Table S8:** List of statistically significant Gene Ontology (GO) terms, KEGG pathways, and tissue‐specific features identified in mouse genome of aged OF versus YF mice.

**Table S9:** List of statistically significant Gene Ontology (GO) terms, KEGG pathways, and tissue‐specific features identified in mouse genome of young YM versus YF mice.

**Table S10:** List of statistically significant Gene Ontology (GO) terms, KEGG pathways identified in the bacteria genome of female OF versus YF mice.

**Table S11:** List of statistically significant Gene Ontology (GO) terms, KEGG pathways expressed lumen fecal exosome (LFE) proteins identified in the bacteria genome of aged male OM versus YM mice.

**Table S12:** List of statistically significant Gene Ontology (GO) terms, KEGG pathways identified in the bacteria genome of female (OF) versus YF mice.

**Table S13:** List of statistically significant differentially expressed lumen fecal exosome (LFE) proteins identified in the bacteria genome of YM versus YF mice.

**Table S14:** List of statistically significant miRNAs in luminal fecal exosome (LFEs) from young and aged mice. OM versus OF.

**Table S15:** List of statistically significant KEGG pathways identified through miRNA target prediction in luminal fecal exosomes (LFEs) from young and aged mice, including the comparisons OM versus OF, OM versus YM, and OF versus YF.

## Data Availability

The datasets generated and/or analyzed during the current study are available from the corresponding author upon reasonable request.
