# Supplementary figures and images for: Gut Luminal Exosomes in Young and Old Mice: Multi‐Omic Characteristics and Regulation of Gut Permeability
Source: Aging Cell. 2026 Mar 26;25(4):e70455. doi: 10.1111/acel.70455 (PMC13140840; doi:10.1111/acel.70455)

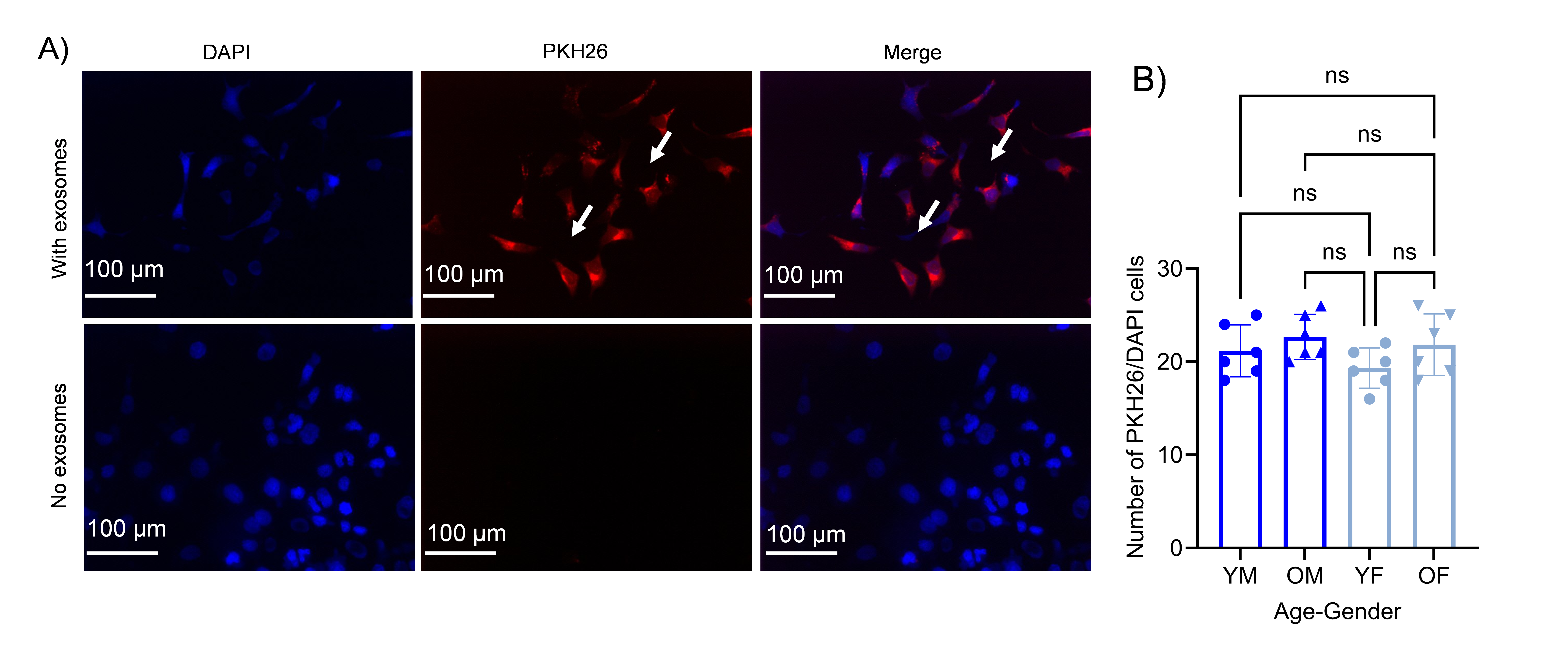

Supplement: Supplementary file 1 — Figure S1: Uptake of luminal fecal exosomes (LFEs) labeled with PKH26 by naïve mouse small intestine cells in vitro. LFEs were labeled with the PKH26 red, fluorescent cell linker for 24 h. Cells were washed and nuclei were stained with DAPI (blue). As a control, PKH26 was added in the absence of LFEs. (A) Fluorescence images of small intestine cells incubated with PKH26‐labeled LFEs (red). Nuclei are shown in blue (DAPI). (B) Semi‐quantitative of LFEs uptakes images. Scale bar, 100 nm. NS indicates no significant. n = 6–8 per group. [file ACEL-25-e70455-s022.tif]

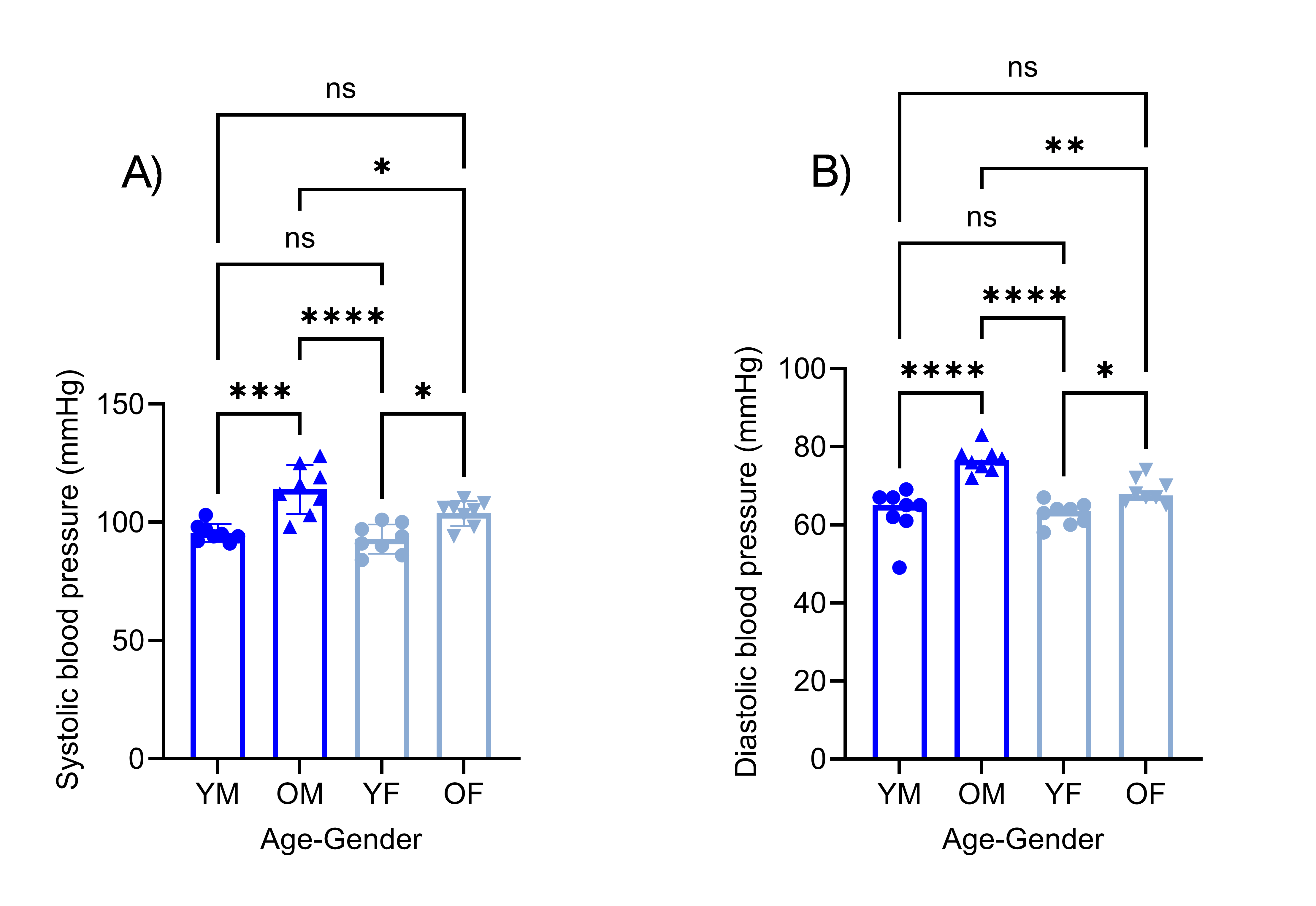

Supplement: Supplementary file 2 — Figure S2: Blood pressure in young and old Mice. Blood pressures were measured across four groups: young males (YM), young females (YF), old males (OM), and old females (OF). (A) systolic blood pressure, and (B) diastolic blood pressure. Statistical comparisons were made using a two‐way ANOVA to assess the effects of age and sex on these parameters. Significant differences in blood pressure were observed between the young and old groups, indicating age‐related physiological changes. Two‐way ANOVA using Tukey's multiple comparisons test. * Indicates p = 0.01, ** p = 0.001, *** p = 0.0001. n = 6–8 per group. [file ACEL-25-e70455-s020.tif]

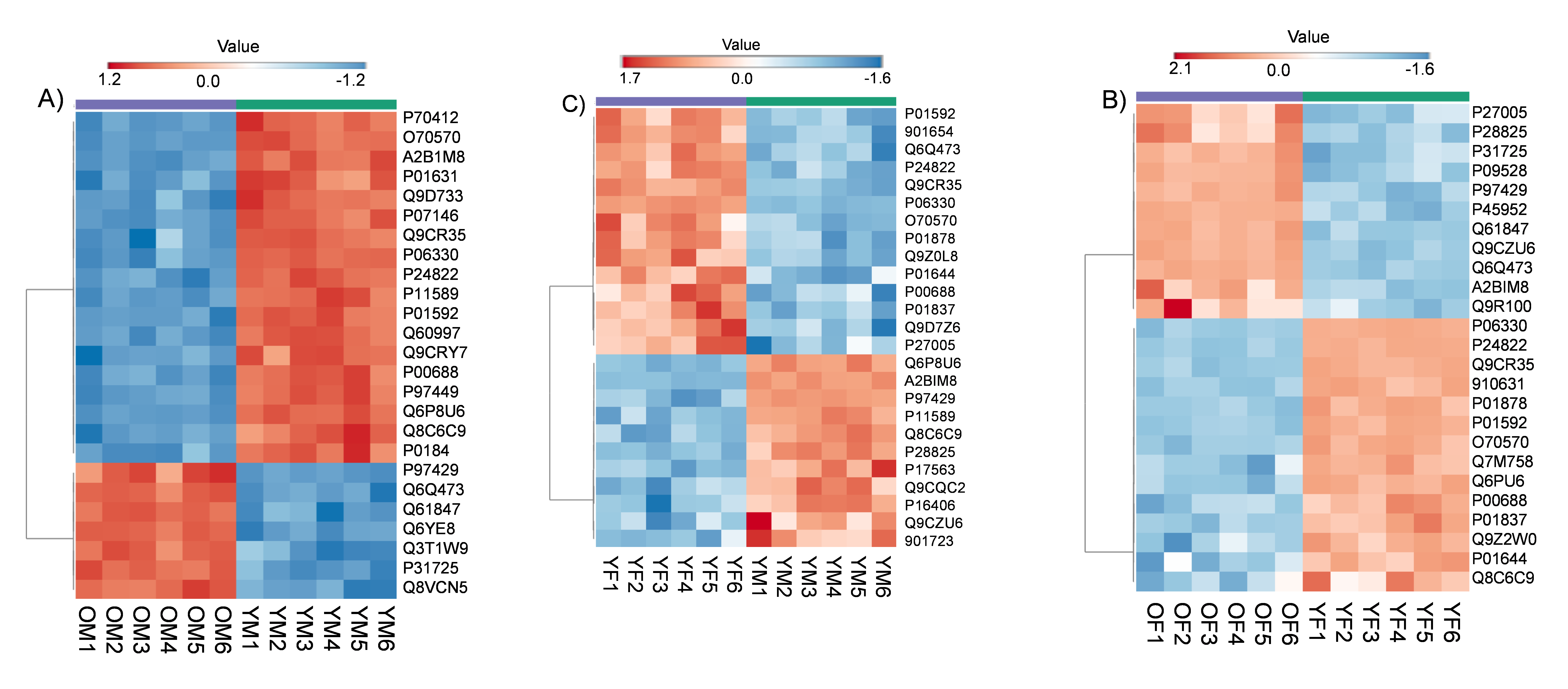

Supplement: Supplementary file 3 — Figure S3: Proteomic profiling of luminal fecal exosomes (LFEs) was performed to characterize host‐derived proteins in young and old mice using liquid chromatography–mass spectrometry (LC–MS). Differentially expressed proteins (DEPs) were identified by searching against the mouse genome database. Heatmap analysis illustrates DEPs for (A) OM versus YM, (B) OF versus YF, and (C) YM versus YF. Red indicates up‐regulated proteins, while blue indicates down‐regulated proteins. Significant protein IDs are shown on the right side of each heatmap. Statistical significance was defined as p < 0.05 with a fold change > 2.0, n = 6 per group. [file ACEL-25-e70455-s001.tif]

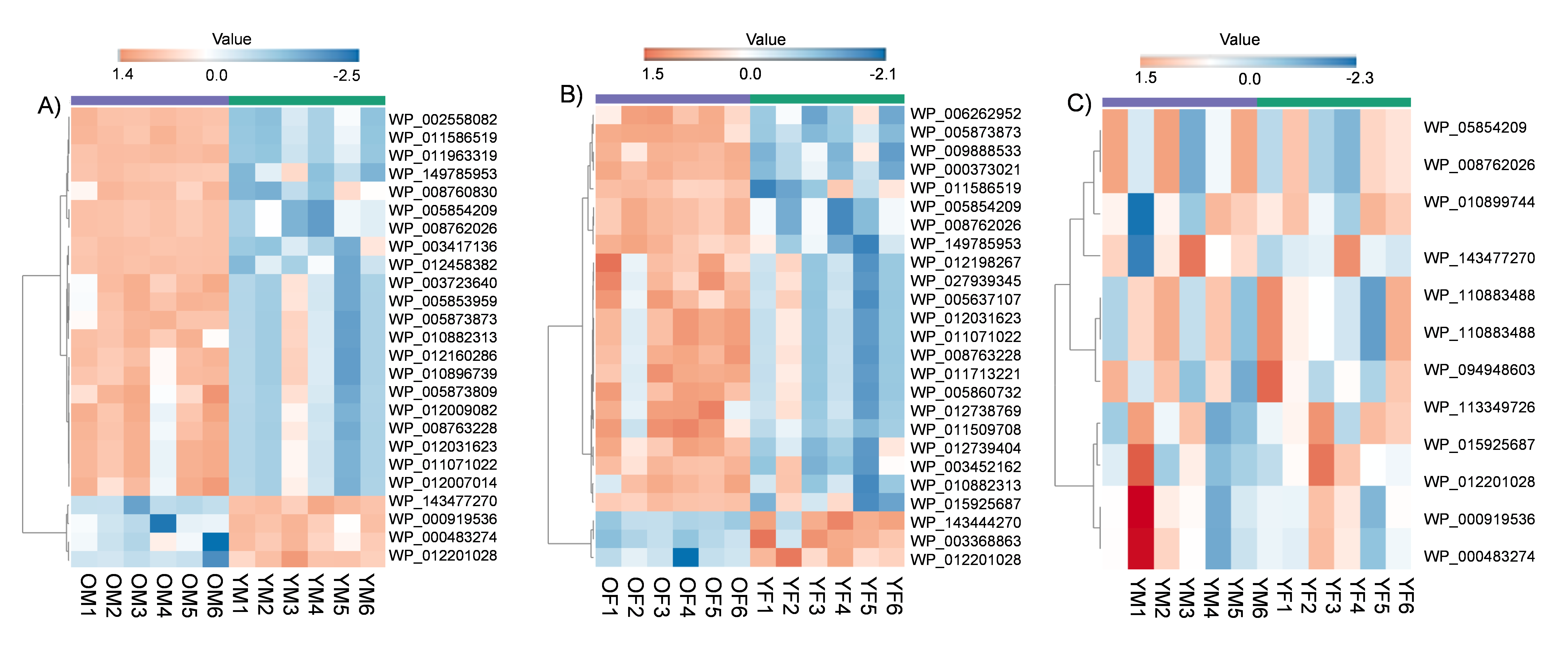

Supplement: Supplementary file 4 — Figure S4: Proteomic profiling of luminal fecal exosome (LFEs) was performed using Liquid Chromatography Mass Spectrometry (LC–MS). Differentially expressed proteins (DEPs) were identified using the search for bacterial genome (microbiota). Heatmap analysis of differentially expressed proteins (DEPs) analysis for (A) OM versus YM, (B) OF versus YF, and (C) YM versus YF. The red color in the heatmap indicates up‐regulation, and the blue color indicates down‐regulation. Significant protein IDs are shown on the right side of each heatmap. Statistical significance was defined as p < 0.05 with a fold change > 2.0, n = 6 per group. [file ACEL-25-e70455-s012.tif]

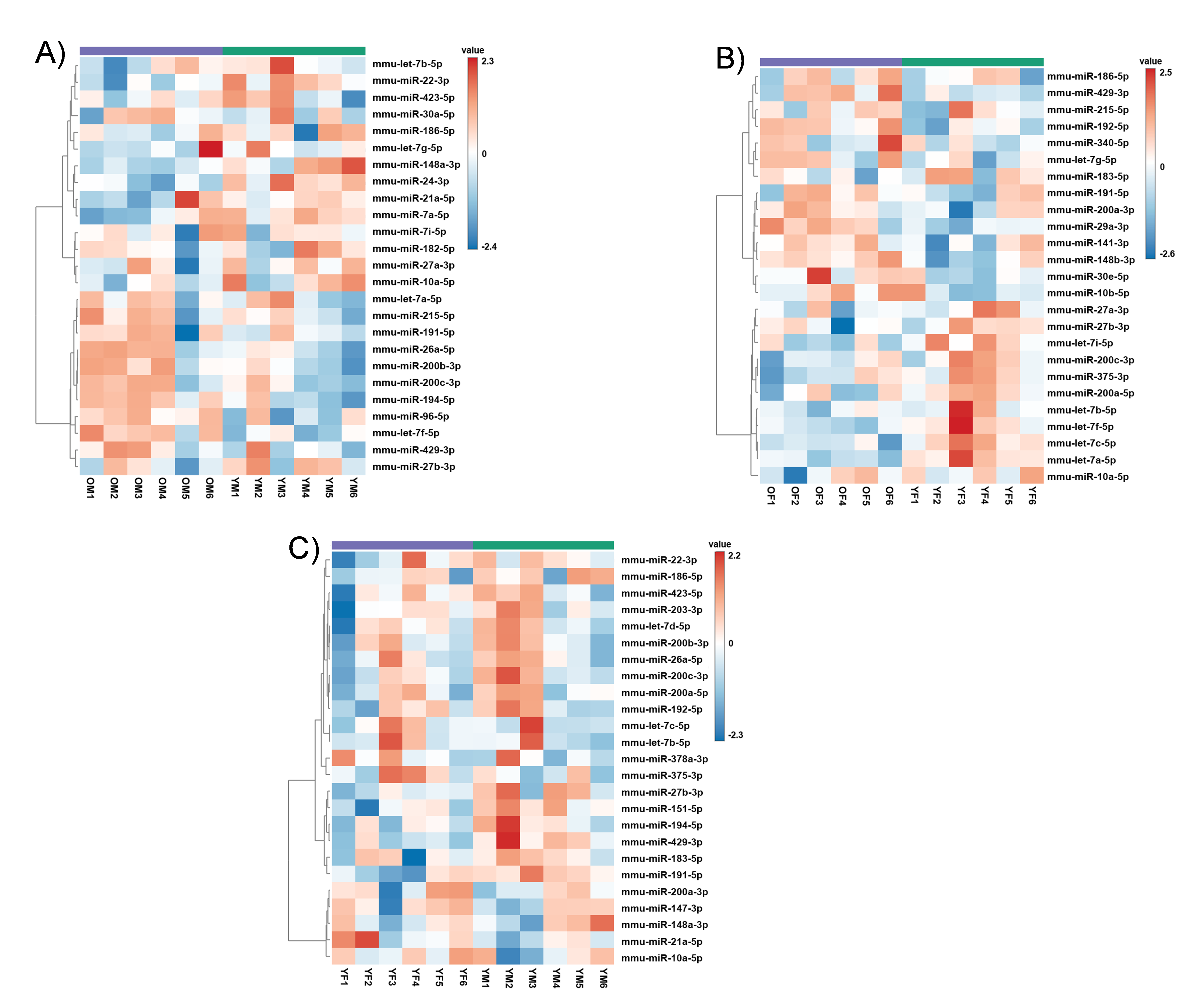

Supplement: Supplementary file 5 — Figure S5: Heatmap of luminal fecal exosomes (LFEs) miRNAs analysis. Heatmap analysis of differentially expressed miRNAs analysis for OM versus YM (A), OF versus YF (B), and YM versus YF (C). The red color in the heatmap indicates up‐regulation, and the blue color indicates down‐regulation. Significant protein IDs are shown on the right side of each heatmap. Statistical significance was defined as p < 0.05 with a fold change > 2.0, n = 6 per group. [file ACEL-25-e70455-s008.tif]

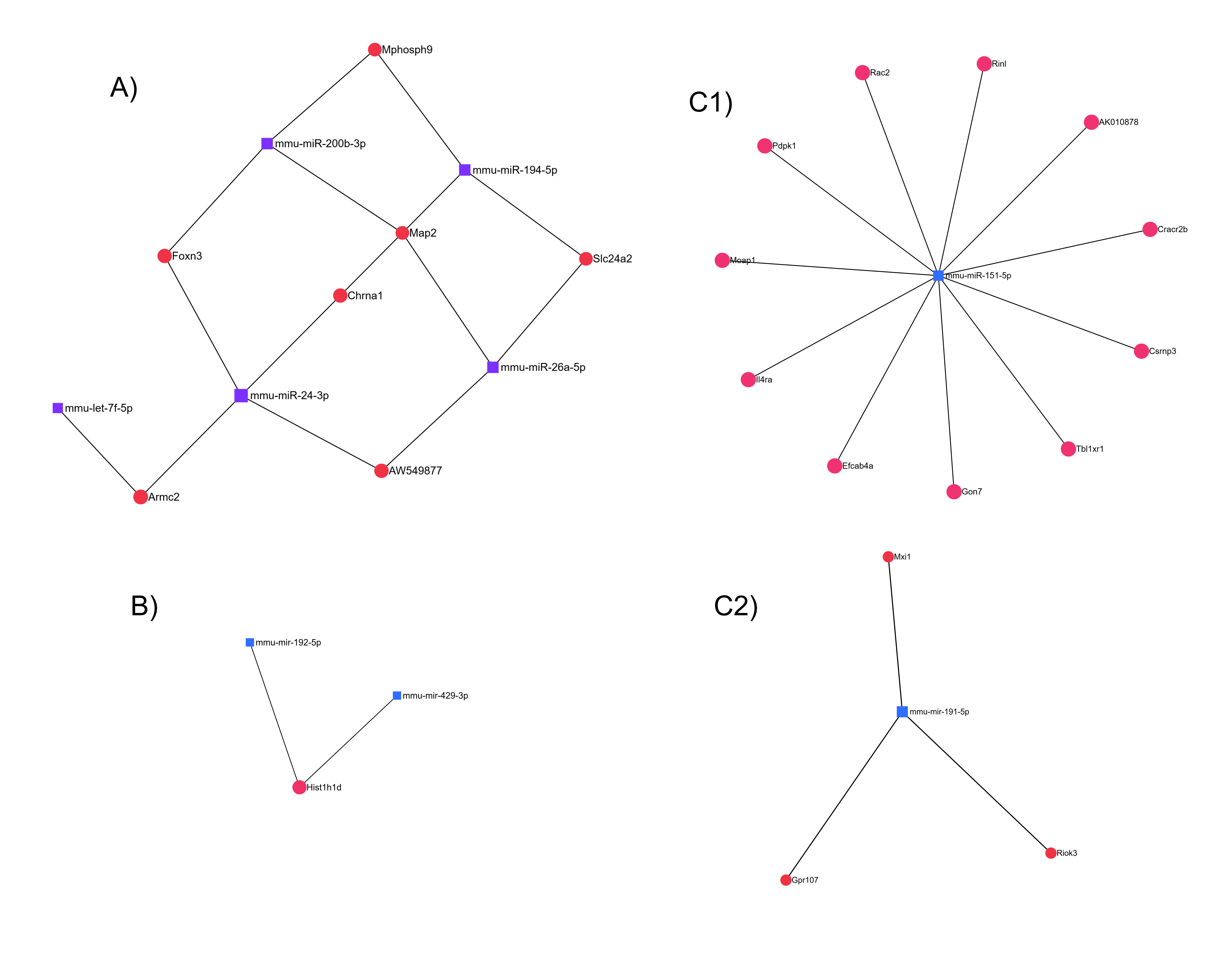

Supplement: Supplementary file 6 — Figure S6: Network analysis of significantly differentially expressed miRNAs across group conditions. (A) miRNA‐mRNA interaction network for OM versus YM shows the minimal target genes for the miRNAs specific to this comparison. (B) Network for OF versus YF highlights the differential interactions in older females compared to younger females. (C) Interaction network for YM versus YF depicts miRNA targets differing between young males and young females. Panel C1 for miRNA‐151‐5p and panel C2 for miRNA 191‐5p. Nodes representing miRNAs are shown in blue, while their corresponding minimum gene targets are shown in red color. Edges signify interactions, and the network structure emphasizes miRNA regulatory roles in different group conditions. [file ACEL-25-e70455-s006.tif]

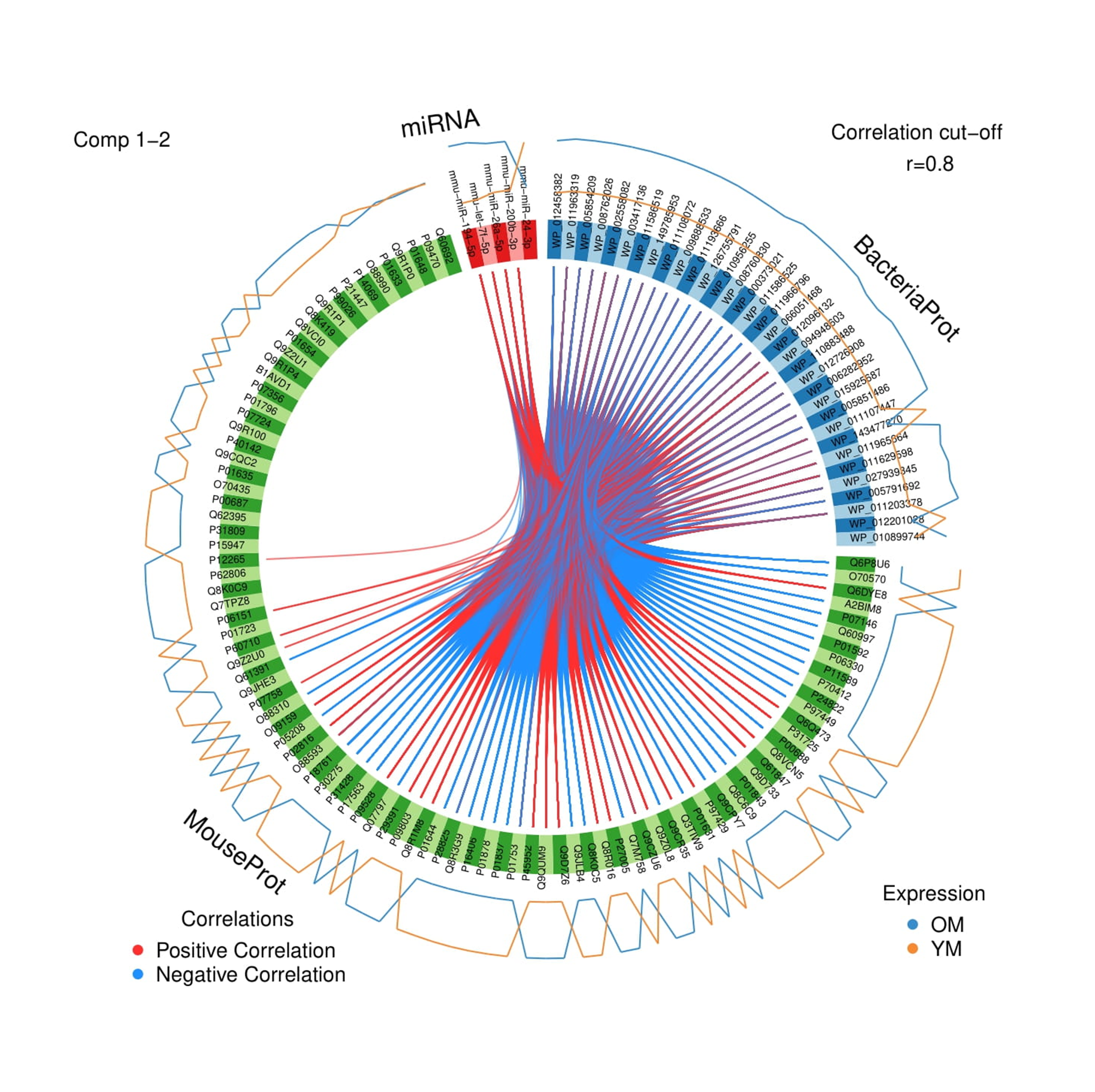

Supplement: Supplementary file 7 — Figure S7: Circle plot analysis integrating miRNA, bacterial genomes, and mouse genome proteins. Outer rings represent distinct data categories: miRNAs (blue), bacterial genomes (green), and mouse genome proteins (yellow). Circle plots between OM versus YM. Edges connecting nodes signify interactions or functional associations derived from bioinformatics analysis. Red color indicates positive correlation, while blue is negative correlations. Lines inside the circle connect pairs of entities with significant correlations (correlation cut‐off at r = 0.8r = 0.8r = 0.8). These connections indicate potential interactions or dependencies between miRNAs, mouse proteins, and bacterial proteins. [file ACEL-25-e70455-s013.tif]

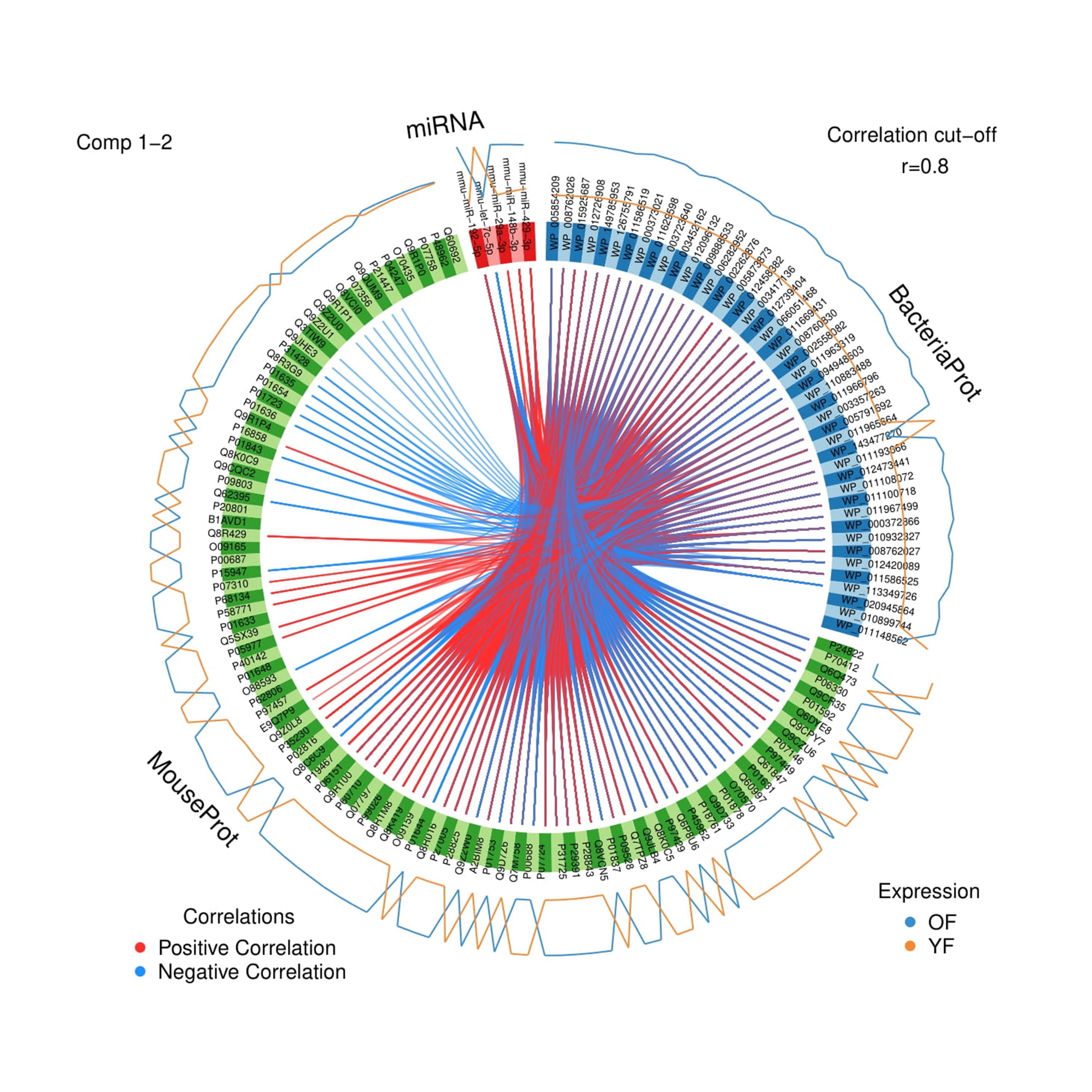

Supplement: Supplementary file 8 — Figure S8: Circle plot analysis integrating miRNA, bacterial genomes, and mouse genome proteins. Outer rings represent distinct data categories: miRNAs (blue), bacterial genomes (green), and mouse genome proteins (yellow). Circle plots between OF versus YF. Edges connecting nodes signify interactions or functional associations derived from bioinformatics analysis. Red color indicates positive correlation, while blue is negative correlations. Lines inside the circle connect pairs of entities with significant correlations (correlation cut‐off at r = 0.8r = 0.8r = 0.8). These connections indicate potential interactions or dependencies between miRNAs, mouse proteins, and bacterial proteins. [file ACEL-25-e70455-s005.tif]

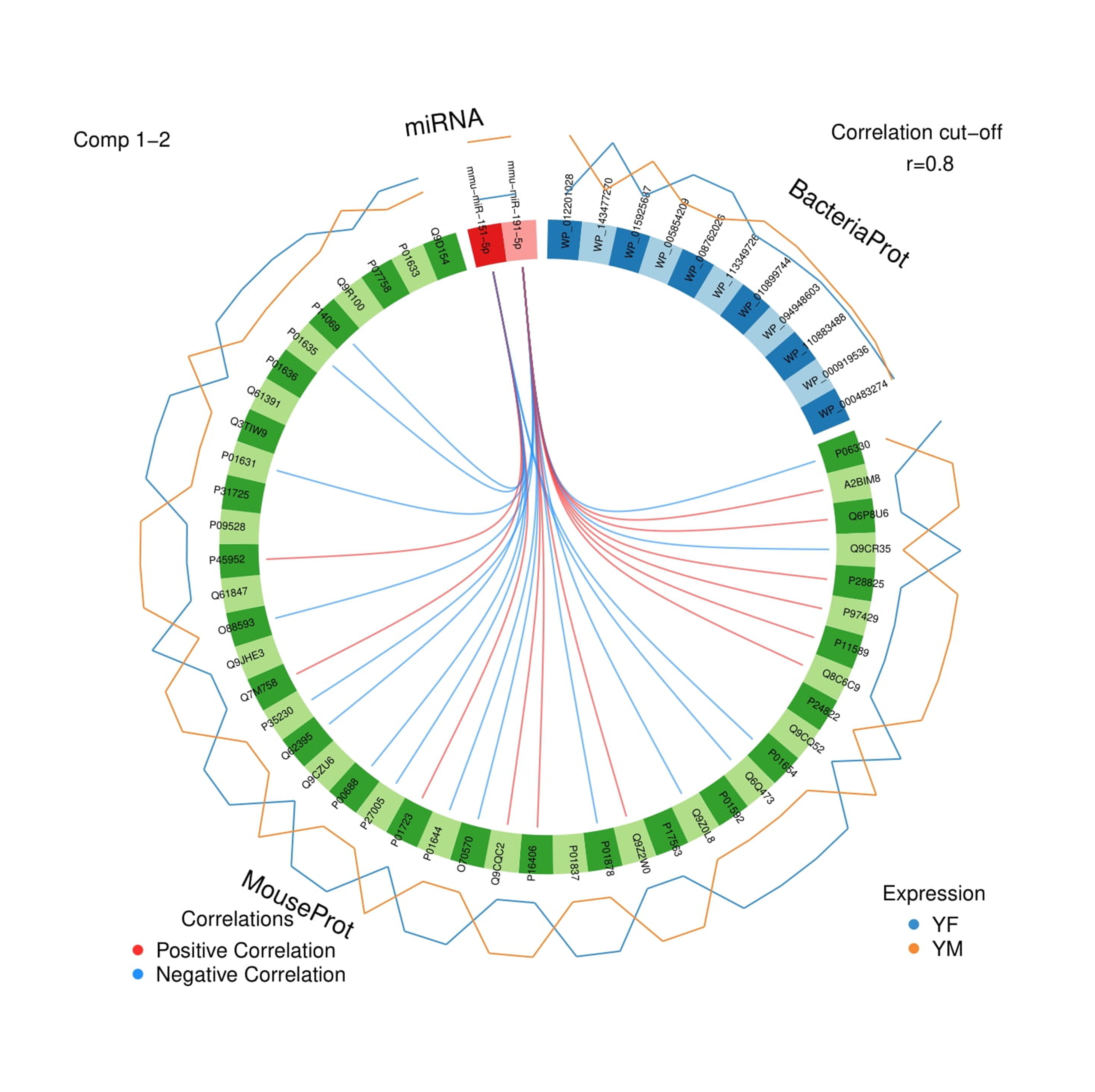

Supplement: Supplementary file 9 — Figure S9: Circle plot analysis integrating miRNA, bacterial genomes, and mouse genome proteins. Outer rings represent distinct data categories: miRNAs (blue), bacterial genomes (green), and mouse genome proteins (yellow). Circle plots between YF versus YM. Edges connecting nodes signify interactions or functional associations derived from bioinformatics analysis. Red color indicates positive correlation, while blue is negative correlations. Lines inside the circle connect pairs of entities with significant correlations (correlation cut‐off at r = 0.8r = 0.8r = 0.8). These connections indicate potential interactions or dependencies between miRNAs, mouse proteins, and bacterial proteins. [file ACEL-25-e70455-s023.tif]
